# Site-Specific *In Situ* Metallization of Wireframe DNA Origami for Reversible Fluorescence Switching

**DOI:** 10.1021/acsnano.6c00622

**Published:** 2026-05-11

**Authors:** Minu Saji, Devanathan Perumal, Tiffany R. Olivera, Henry Wisniewski, Shaden Salim, Fei Zhang

**Affiliations:** Department of Chemistry, 67206 Rutgers University, Newark, New Jersey 07102, United States

**Keywords:** DNA origami, gold, *in situ* metallization, nanoparticles, Cy3 dye, fluorescence quenching, strand displacement reaction

## Abstract

The addressability of DNA origami nanostructures enables the site-specific positioning of functional materials with nanometer-scale precision. DNA origami-templated gold nanoparticles have attracted broad interest due to their tunable optical and electronic properties. In this study, we develop a bottom-up nanofabrication strategy that integrates a rationally designed chemical handle that contains an iodobenzene derivative into a wireframe DNA origami template to direct the capture and *in situ* reduction of gold ions, achieving site-specific metallization. We then incorporate a fluorescent Cy3 dye into the metallized origami template to show the distance-dependent fluorescence quenching via energy transfer. As a proof of concept, we also demonstrate the dynamic programmability of the hybrid system to remove the fluorophore by using a strand displacement reaction, resulting in complete fluorescence recovery.

## Graphical abstract

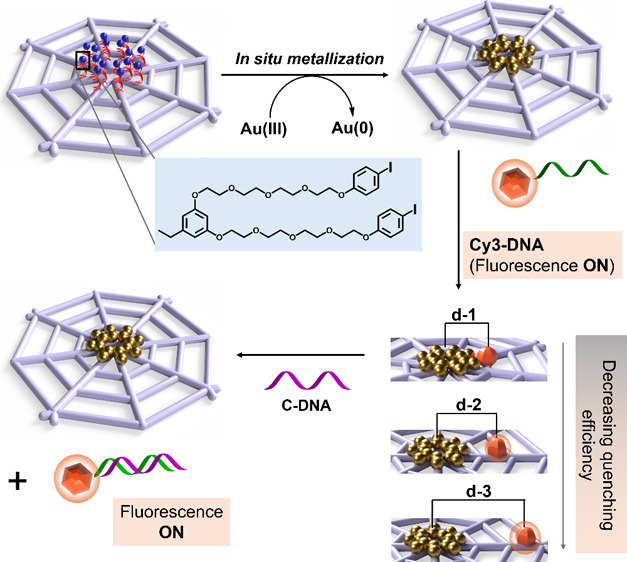

## Introduction

The optical, electronic, and magnetic properties of inorganic nanomaterials are strongly dependent on their composition, size, shape, and spatial arrangement.^1^ As a result, substantial research efforts have focused on the development of inorganic nanoparticles with customizable features and narrow size distribution for a wide range of applications, including nanophotonics,^2^ electronic nanodevices,^3,4^ catalytic systems,^5–7^ and biomedical science.^8–10^ Due to its intrinsic addressability and nanoscale tunability, DNA origami has emerged as a promising template for the bottom-up nanofabrication of inorganic materials, such as silica,^11–13^ calcium phosphate,^14^ and metal and metal oxide nanoparticles.^15–19^ Recent work has also demonstrated the DNA-templated assembly of three-dimensional (3D) metal, metal oxide, and semiconductor frameworks,^20^ as well as the homogeneous inorganic coating of DNA origami crystals using techniques such as atomic layer deposition.^21^ These origami-templated approaches thus highlight the potential of DNA nanostructures to direct the construction of complex nanoarchitectures with high spatial precision, structural uniformity, and precise morphological control.^17,22^

Two main strategies have been employed to direct DNA origami-templated metallization. The first method relies on conjugating an externally synthesized nanoparticle to the origami through hybridization of DNA strands attached to the nanoparticle with complementary strands protruding from the origami.^23–26^ These nanoparticles can serve as seeds for further growth by the site-specific reduction of metal salts on them.^27–29^ The second strategy involves engineering a nucleation site to condense metal precursors, which are subsequently reduced into the corresponding nanoparticles using specific reducing agents.^15,16,30–33^ Many such strategies have focused on gold nanoparticles due to their distinctive characteristics like localized surface plasmon resonance,^34,35^ tunable optical properties,^36–38^ and ease of surface functionalization.^39–42^ Presynthesized gold nanoparticles have been attached to DNA nanostructures through DNA hybridization to form highly ordered plasmonic assemblies.^43–47^ However, as the complexity of the DNA architectures increases, this strategy may suffer from steric hindrance to the diffusion of external nanoparticles. For example, in the system of complex 3D DNA lattices, inserting nanoparticles into the internal binding cavities can be very difficult or even impossible, leading to reduced site occupancy and low assembly efficiency. *In situ* nanoparticle synthesis, therefore, offers an alternative strategy by creating a localized chemical environment for the nucleation and reduction of gold precursors directly on the origami template, potentially improving site-selectivity and assembly yield for the fabrication of complex plasmonic systems. Previous studies have explored the generation of gold nanoparticles mediated by halide ions.^48,49^ Pigliacelli et al. reported C–I bond activation on phenylalanine iodobenzene for the synthesis of gold nanoparticles in 2019.^50^ However, such bond-activation chemistry has not been explored as an orthogonal strategy for directing the *in situ* synthesis of gold nanoparticles on DNA origami templates.

Motivated by this gap, we developed an iodide-mediated metallization strategy using a homemade iodobenzene derivative conjugated to DNA (**Iodo-DNA**) to achieve the site-specific growth of gold nanoclusters of controlled size directly on a wireframe DNA origami scaffold (Figure 1a). We then constructed a programmable fluorescent system by incorporating a Cy3 fluorophore into the metallized origami template and demonstrated distance-dependent fluorescence quenching (turn “OFF”) via energy transfer to the gold nanoclusters. Finally, we employed the toehold-mediated strand displacement reaction (TMSDR)^51,52^ to remove the fluorophore from the origami system, thereby effectively turning the fluorescence back “ON” (Figure 1b). A wireframe origami nanostructure modified with externally synthesized gold nanoparticle-DNA conjugates was used as a reference system to compare the metallization efficiency, proximity-induced fluorescence quenching, and the subsequent TMSDR-mediated fluorescence recovery with the *in situ* metallized nanoswitch (Figure 1c).

**Figure 1:**
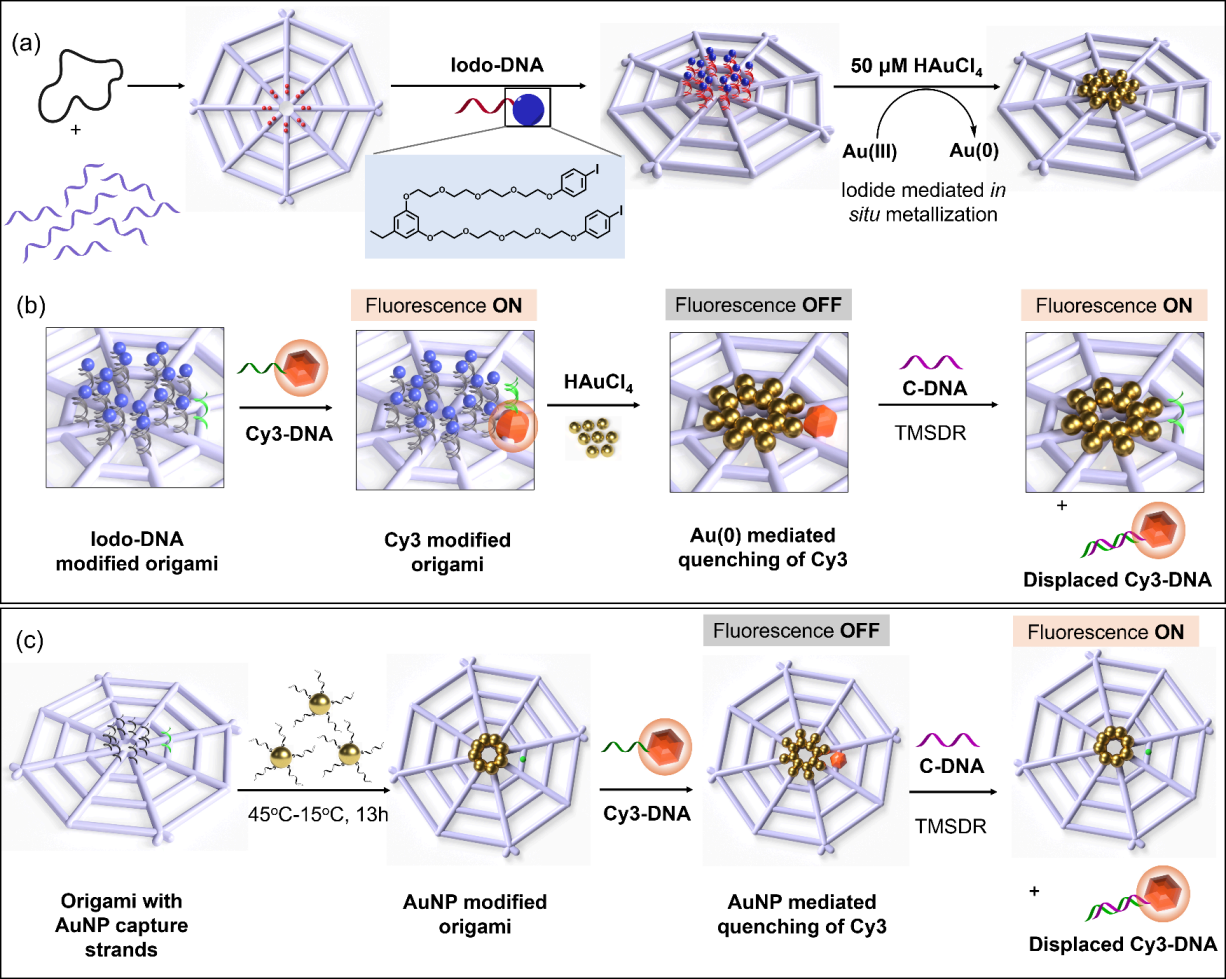
Schematic illustration of (a) wireframe DNA origami template with 16 protruding capture strands at the center for **Iodo-DNA** hybridization and the subsequent site-specific metallization, (b) conjugation of **Cy3-DNA** to **Iodo-DNA**-modified wireframe origami (fluorescence “ON”), metallization of the fluorescent origami template leading to quenching of Cy3 fluorescence (turn “OFF”), and the displacement of **Cy3-DNA** from the template by hybridization with an invader strand, **C-DNA**, by TMSDR for fluorescence recovery (turn “ON”), and (c) wireframe origami design with eight protruding capture strands for AuNP hybridization as a reference system, fluorescence turn “OFF” via **Cy3-DNA** conjugation to metallized origami and subsequent fluorescence recovery (turn “ON”) by displacement of **Cy3-DNA** from the template through TMSDR.

## Results and Discussion

### Synthesis and Design

We first designed an octagonal, spider-web-shaped wireframe DNA origami nanostructure with a diameter of 145 nm and a thickness of 2 nm by folding a long M13mp18 scaffold DNA (7249 bases) using short staple strands. This origami template was designed to have 16 single-stranded protruding capture strands distributed among the eight arms at the innermost layer. We then designed and synthesized a dendrimer containing two equivalents of iodobenzene (**2-IB**) to act as the reducing agent for the conversion of Au­(III) to Au(0) on the origami template (Figure 2a). The detailed synthesis schemes are included in the Supporting Information (Scheme 1, Synthesis Schemes). The ability of this custom-synthesized molecule to form gold nanoclusters was evaluated in various buffer systems, including HEPES-Mg^2+^ and TAE-Mg^2+^. A visible color change from colorless to purple was observed when the molecule was incubated with HAuCl_4_ for 4 h in HEPES buffer (Figure S1). The formation of gold nanoclusters was confirmed by the presence of a characteristic plasmonic absorbance peak in the 500–800 nm range when 100 μM of **2-IB** was reacted with 500 μM of Au­(III). No plasmonic band was observed for **2-IB** or Au­(III) alone, indicating that nanocluster formation requires both components (Figure 2b). In this system, HEPES is expected to function as both a stabilizing agent and a weak reducing agent,^53,54^ which generates the initial reduced gold species that result in the activation of the C–I bond of **2-IB**. This activation may proceed through several mechanisms highlighted in the previous literature.^50,55,56^ The activated C–I bonds are then expected to promote the generation of Au(0) species and work as nucleation sites for the formation of gold nanoparticles. Our experimental data indicate the need for both HEPES and **2-IB** for effective reduction and nucleation during gold nanoparticle formation.

**Figure 2:**
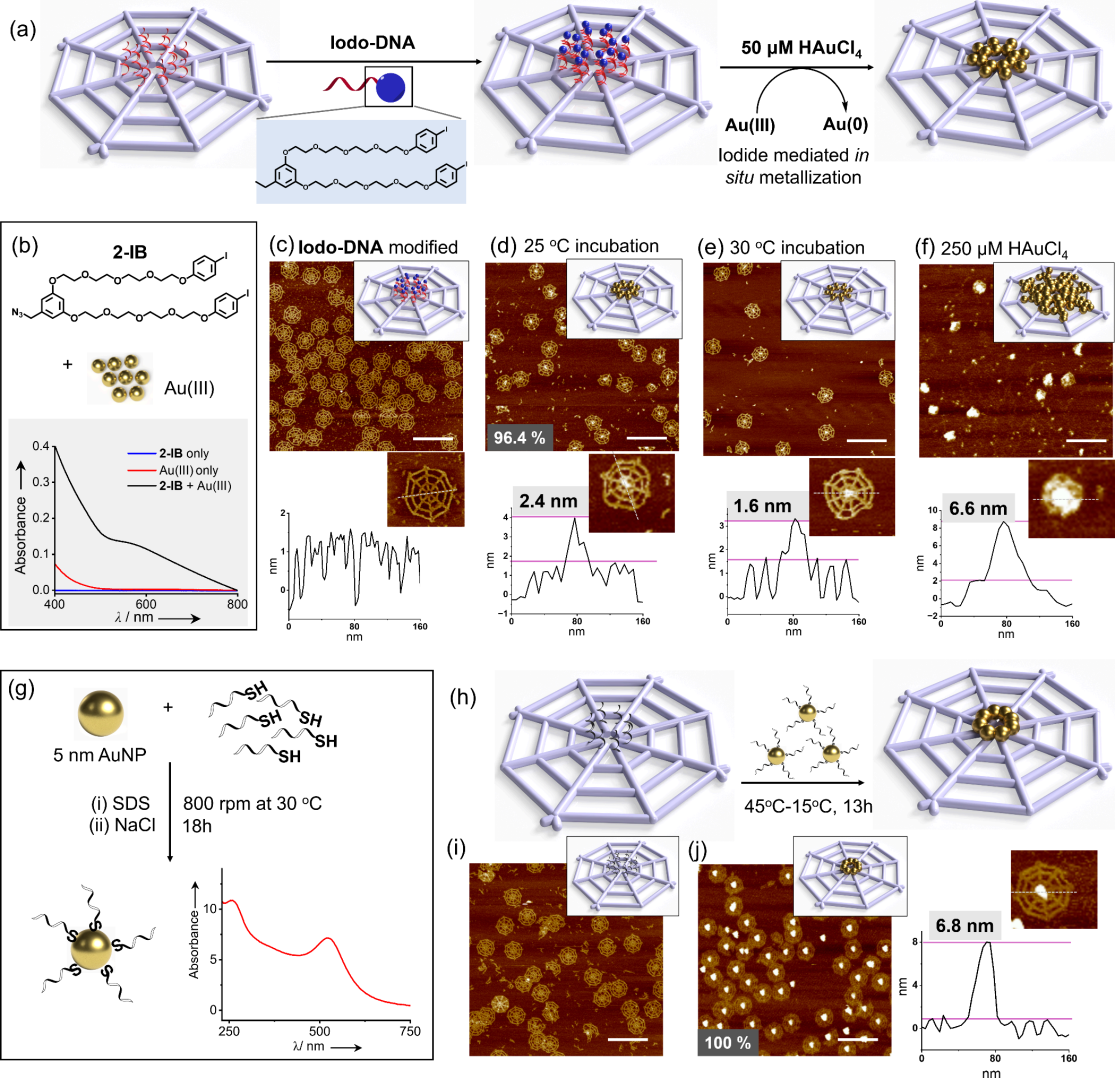
(a) Schematic illustration showing the mechanism of **Iodo-DNA** conjugation to the wireframe origami template to direct site-specific gold nanocluster formation at the center of the origami. (b) UV–vis absorbance spectrum showing the appearance of the plasmonic band (black line) when 100 μM of **2-IB** was treated with 500 μM of HAuCl4 in 1 mM HEPES-12.5 mM Mg^2+^ buffer; the red and blue plots show the control experiments using 500 μM of HAuCl4 and 100 μM of 2-IB, respectively, in the same buffer system. The plasmonic band is not visible in these control experiments. The AFM image and height analysis of (c) origami template that has been modified with **Iodo-DNA**, which shows a uniform thickness of ∼1.5 nm without any visible height increase at the modification site, (d) **Iodo-DNA**-modified origami treated with 50 μM of HAuCl4 and shaken at 25 °C for 5 h, showing a height increase of 2.4 nm at the modification site; 96.4% of the origami counted showed successful metallization (*N* = 166), (e) **Iodo-DNA**-modified origami treated with 50 μM of HAuCl4 and incubated at 30 °C for 5 h, showing a smaller height increase of 1.6 nm at the modification site, (f) **Iodo-DNA**-modified origami treated with 250 μM of HAuCl4 and shaken at 25 °C for 10 min, showing metal overgrowth with a large height increase of 6.6 nm at the modification site (scale bar: 300 nm). (g) Schematic illustration of the conjugation of 5 nm AuNPs to thiol-modified DNA and the corresponding UV–vis spectrum showing the characteristic absorbance of DNA at 260 nm and those of AuNPs at 520 nm. (h) Schematic illustration showing the conjugation of AuNP-DNA to the origami template by hybridizing with eight protruding capture strands. (i) AFM image of the wireframe origami template with protruding capture strands (scale bar: 300 nm). (j) AFM image showing the successful conjugation of AuNPs to the template with 100% yield (*N* = 245, scale bar: 300 nm) and AFM height analysis showing a height increase of 6.8 nm at the modification site.

This **2-IB** derivative was subsequently conjugated to a dibenzocyclooctyne-tetraethylene glycol (DBCO–TEG)-modified single-stranded DNA (18 nt) via Click reaction in a 1:1 water/DMSO reaction mixture. The resulting **2-IB**-modified DNA (**Iodo-DNA**) was purified by HPLC and characterized by MALDI-TOF spectrometry (Figure S2).

### Site-Specific *In Situ* Reduction of Au­(III) on a Wireframe DNA Origami Template

After confirming the successful reduction of gold salts into nanoclusters facilitated by the **2-IB** derivative molecule, the wireframe DNA origami was modified with **Iodo-DNA** in the regions with the 16 single-stranded capture strands (Table S1 and Figure S3). A mixture of 3 nM : 6 nM scaffold/staple DNA strands was annealed in 1 mM HEPES buffer containing 12.5 mM Mg^2+^. Atomic force microscopic (AFM) images were used to show the assembly of the origami nanostructures, and no significant height increase was observed at the modification site (Figure 2c and Figure S4). This hybrid origami was then treated with 50 μM HAuCl_4_ without any additional purification and was shaken at 25 °C for 5 h. AFM analysis of an individual representative origami shows a vertical height increase of 2.4 nm in the innermost location of the wireframe origami, which was designed to capture the **Iodo-DNA**. 96.4% of all the origami counted showed successful metallization at the predesigned location (Figure 2d and Figures S5 and S8). Interestingly, the nanoclusters remained localized to the central core of the wireframe origami even after 24 h, without any further overgrowth, indicating the completion of the reduction reaction and the formation of stable nanoclusters (Figure S10). Negative control experiments were performed using wireframe origami modified with double-stranded DNA instead of the **Iodo-DNA** at the prescribed sites. No significant metallization was observed in this case, showing that the **Iodo-DNA** is essential for metal growth (Figure S6). Next, we studied the effect of tuning the temperature on the metallization reaction. Upon incubating the modified origami with Au­(III) salts at a slightly elevated temperature of 30 °C for 5 h, slightly smaller and more uniform metal growth was observed at the center of the origami compared to the reaction at 25 °C. This was characterized by AFM height analysis, which showed a relatively lower height increase of 1.6 nm at the metallized location (Figure 2e and Figures S7 and S11). This suggests that careful temperature tuning can help control the rate of nucleation and the final nanoparticle size. Finally, we also demonstrated the effect of treating origami with a very high concentration of Au­(III) salts (250 μM). AFM analysis revealed complete coverage of the origami template via metal overgrowth after just 10 min of reaction, indicating a concentration-dependent increase in the reaction rate (Figure 2f and Figure S12). The metallization was found to be the highest in the central core modified with **Iodo-DNA**, which then proceeded to grow toward the outer layers of the wireframe, forming a thinner gold layer covering the rest of the template. Metal nanocluster growth with a vertical height of 6.6 nm was observed at the central location of the origami from AFM analysis (Figure 2f). We also modified the origami design to possess different **Iodo-DNA** handle densities to study how this affects the yield and particle size as shown in Figure S13. AFM analysis indicates an increase in nanocluster size and metallization yield with an increase in the density of **Iodo-DNA** handles, pointing to the programmability of this metallization strategy.

### Gold Nanoparticle Attachment on the Origami Template

After demonstrating the successful site-specific gold growth on the origami template using the **Iodo-DNA** conjugate, a reference system was engineered by incorporating externally synthesized gold nanoparticles (AuNPs) into the origami template to compare its behavior with our *in situ* metallized system. For this, we first functionalized commercially purchased 5 nm diameter AuNPs with thiol-modified DNA (20 nt with a T-10 spacer) via Au–S bond formation (see details in the Materials and Methods section). These AuNPs decorated with DNA strands showed the characteristic absorbance peak of DNA at 260 nm and those of AuNPs at 520 nm (Figure 2g). The wireframe origami was designed to include eight protruding single-stranded capture DNA (10 nt), which was complementary to the DNA strands modified on the AuNPs. The unpurified origami template (3 nM) was then treated with 48 nM AuNPs and gradually cooled down from 45 to 15 °C over 13 h to enable successful AuNP functionalization (Figure 2h). AFM analysis of the origami template with capture strands did not show any visible height increase (Figure 2i), while those modified with AuNPs displayed a growth at the inner core with a height of 6.8 nm and a diameter of ∼40 nm, indicating the capture of multiple AuNPs per origami template at an essentially quantitative yield (∼100%) for functionalization of DNA origami templates (Figure 2j and Figures S15 and S16).

### Time-Dependent Quenching of Cy3 Fluorescence by *In Situ* Gold Nanocluster Growth

The ability of gold to quench the fluorescence of Cy3 dye via energy transfer from Cy3 (donor) to the metal is of great importance in the design of gold-based biosensors for the detection of specific analytes.^57,58^ Building on our site-specific gold nanocluster deposition strategy, we next leveraged the programmability of DNA origami to position fluorophores with nanometer precision and construct a fluorescence-based nanoswitch capable of reversible “ON” and “OFF” states. As an initial demonstration, we incorporated **Cy3-DNA** proximal to the **Iodo-DNA** modification site at the inner core of the wireframe template, resulting in a fluorescent “ON” state (Figure 3a). The **Cy3-DNA** conjugate was designed in such a way that the Cy3 dye was positioned in the same plane as the origami template to enable a more favorable conformation for successful gold-Cy3 interaction. The fluorophore-modified origami was then treated with 50 μM HAuCl_4_ to induce the controlled formation of gold nanoclusters at the prescribed location (Figure 3b), consistent with the metallization conditions shown in Figure 2d. Fluorescence spectroscopy was used to analyze the time-dependent quenching of Cy3 fluorescence (turn “OFF”) from the point of Au­(III) addition (0 h) up to 5 h (Figure 3c and Figure S17). At an excitation wavelength of 520 nm, the majority of the fluorescence quenching occurred within the first hour, indicating that the reduction reaction of Au­(III) to Au(0) proceeded to near completion on this time scale.

**Figure 3:**
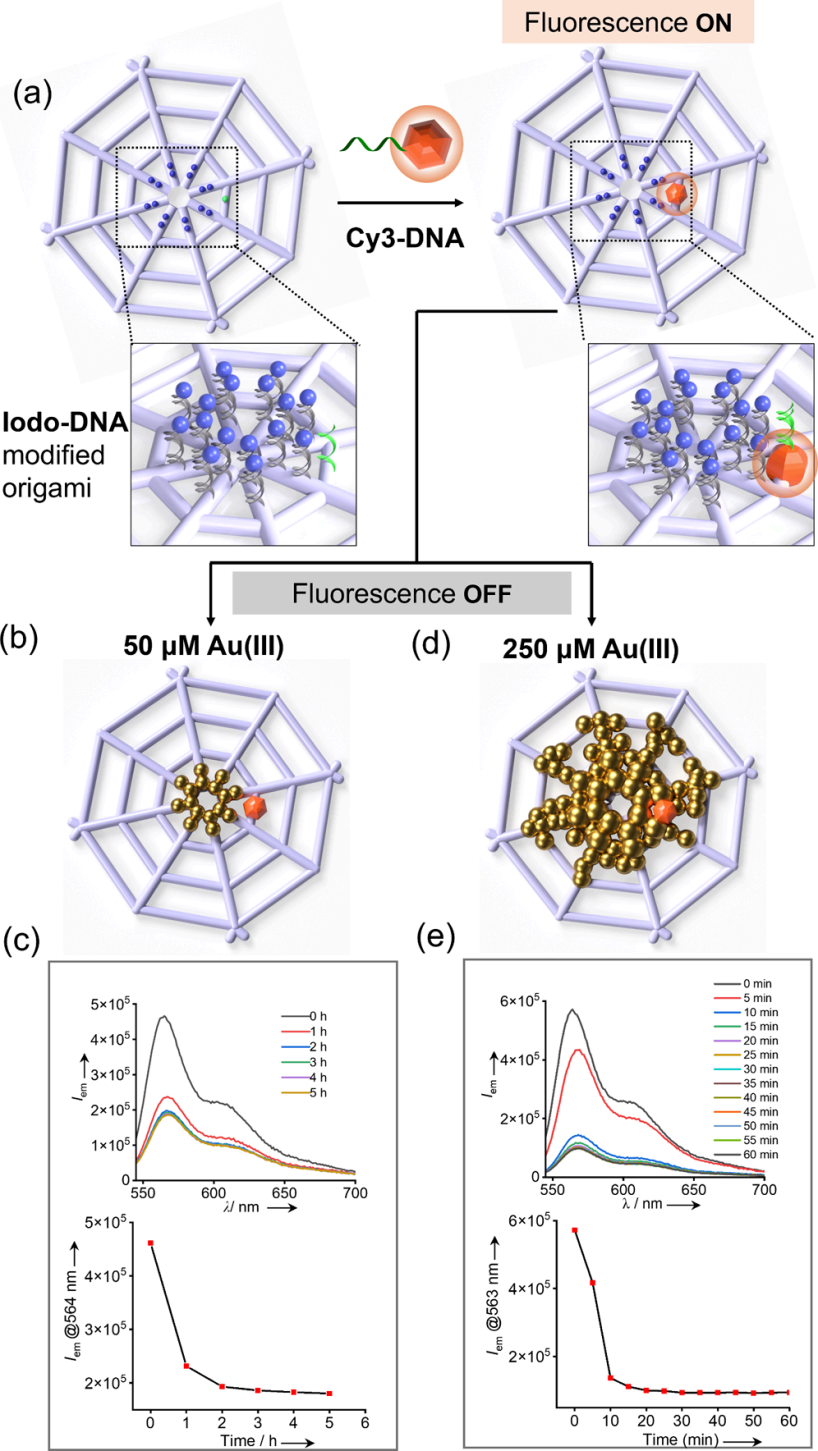
Time-dependent quenching of Cy3 fluorescence by origami-templated gold nanoclusters. (a) Schematic illustration of conjugation of **Cy3-DNA** to **Iodo-DNA**-modified wireframe origami template for fluorescence turn “ON”. (b) Fluorescence turn “OFF” by treatment of the origami template with 50 μM Au­(III) for controlled nanocluster formation. (c) Time-dependent quenching of Cy3 fluorescence up to 5 h after the addition of 50 μM Au­(III) showing that much of the quenching happens within the first hour. (d) Fluorescence turn “OFF” by treatment of the origami template with 250 μM Au­(III) for gold overgrowth. (e) Time-dependent quenching of Cy3 fluorescence up to 60 min after the addition of 250 μM Au­(III), showing that much of the quenching happens within the first 10 min.

To examine the effect of accelerated metallization, an analogous Cy3-modified origami template was treated with 250 μM HAuCl_4_ to induce metal overgrowth (Figure 3d), as previously demonstrated in Figure 2f. The sample was excited at a wavelength of 520 nm, and the fluorescence quenching was monitored for 1 h after the start of the reaction. As expected, much of the Cy3 fluorescence was quenched within the first 10 min after the addition of the Au­(III) salt (Figure 3e and Figure S17). These results demonstrate that increased Au­(III) concentration accelerates nanocluster formation, leading to rapid and nearly complete fluorescence quenching.

### Distance-Dependent Quenching of Cy3 Fluorescence by *In Situ* Gold Nanocluster Growth

The programmability of DNA origami enables precise control over the spatial separation between fluorophores and metal nanoclusters. The quenching efficiency is expected to be the highest when Cy3 is positioned closest to the gold nanoclusters and to decrease with increasing separation distance. In addition, the quenching efficiency also depends on the size and morphology of the gold nanoclusters. Motivated by this, we compared the distance-dependent quenching efficiency of the *in situ* generated gold nanoclusters with that of the externally synthesized AuNPs conjugated to the wireframe origami. We first modified our wireframe template (with **Iodo-DNA**) to incorporate the Cy3 dye at three defined distances from the center point of the origami: **d-1** (nearest), **d-2** (intermediate), and **d-3** (farthest). Fluorescence quenching efficiencies were compared after 5 h of Au­(III) addition (Figure 4a–c and Figure S19). For these *in situ* metallized templates, the fluorescence quenching (black line) decreased compared with the unmetallized control (red line) as the fluorophore was placed farther away from the center. The maximum quenching was observed when the fluorophore was placed at **d-1** (∼23.5 nm), where the normalized fluorescence intensity decreased from 1 to 0.47 (±0.05, *N* = 3). At the intermediate distance of **d-2** (∼36.5 nm), the fluorescence was quenched from 1 to 0.64 (±0.05, *N* = 3), while positioning Cy3 at the farthest distance **d-3** (∼55.5 nm) resulted in a smaller reduction from 1 to 0.83 (±0.03, *N* = 3). Hence, the quenching efficiency follows the expected order **d-1** > **d-2** > **d-3** (Figure 4d). Negative control fluorescence experiments were performed on the Cy3-origami system to confirm that there is no significant quenching of emission intensity in the absence of **Iodo-DNA** under the same experimental conditions (Figure S18).

**Figure 4:**
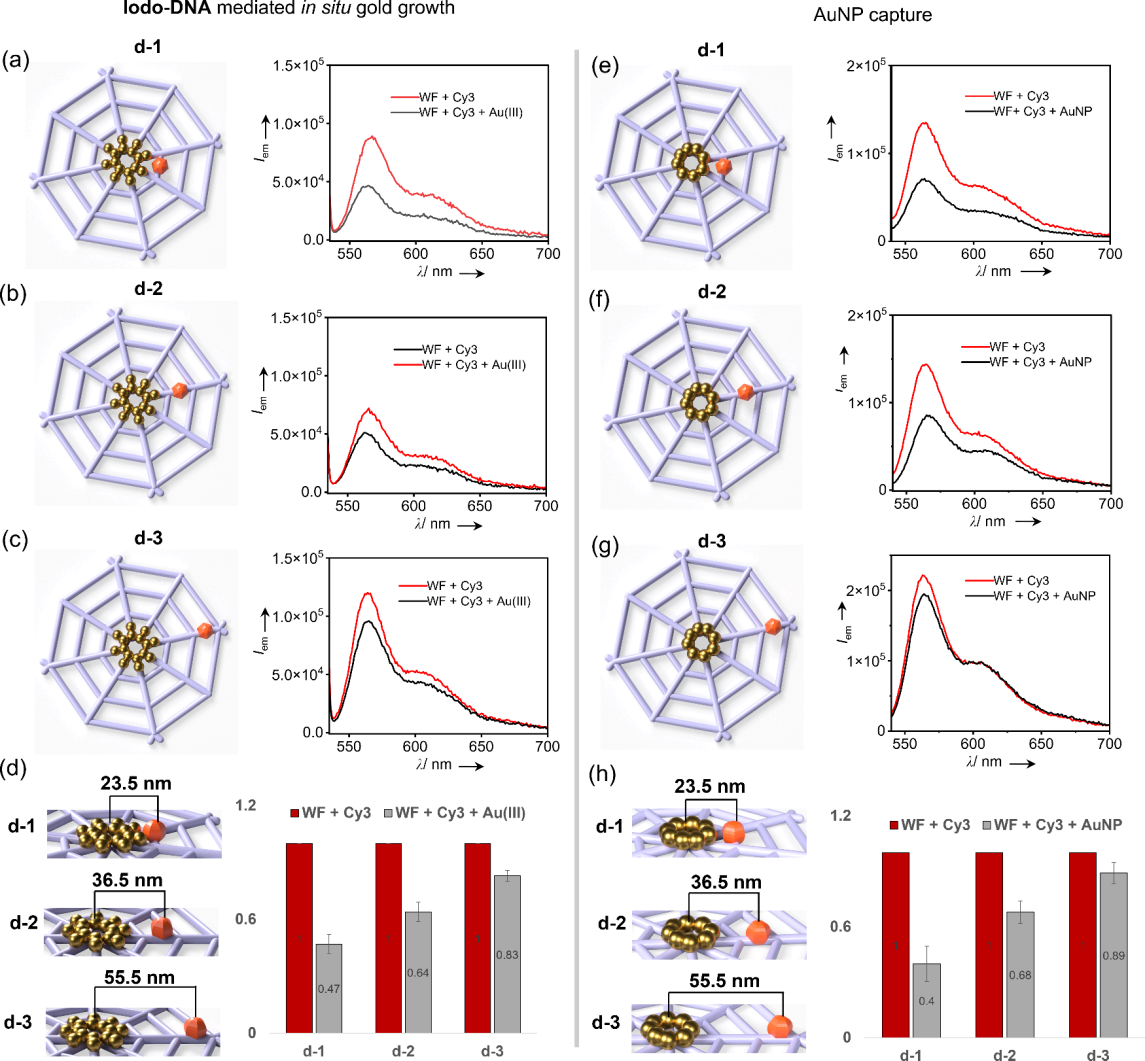
Distance-dependent quenching of Cy3 fluorescence by wireframe origami (WF)-templated gold growth. Schematic illustration and emission spectrum of (a) Cy3 dye placed at distance **d-1**, (b) Cy3 dye placed at distance **d-2**, and (c) Cy3 dye placed at distance **d-3**, showing the quenching of Cy3 fluorescence after an *in situ* metallization reaction. (d) Comparison of quenching efficiencies at distances **d-1**, **d-2**, and **d-3**; the normalized fluorescence intensity was quenched from 1 to 0.47 ± 0.05 at **d-1**, from 1 to 0.64 ± 0.05 at distance **d-2**, and from 1 to 0.83 ± 0.03 at distance d-3, therefore following the efficiency order **d-1** > **d-2** > **d-3**. Schematic illustration and emission spectrum of (e) Cy3 dye placed at distance **d-1**, (f) Cy3 dye placed at distance **d-2**, and (g) Cy3 dye placed at distance **d-3**, showing the quenching of Cy3 fluorescence after 5 nm AuNP conjugation to the origami template. (h) Comparison of quenching efficiencies at distances **d-1**, **d-2**, and **d-3**; the normalized fluorescence intensity was quenched from 1 to 0.40 ± 0.10 at **d-1**, from 1 to 0.68 ± 0.06 at distance **d-2**, and from 1 to 0.89 ± 0.06 at distance **d-3**, therefore following the efficiency order **d-1** > **d-2** > **d-3**. *N* = 3 for all the experiments, and the normalized emission intensity is reported as mean ± SD.

Next, we designed three wireframe templates modified with externally synthesized 5 nm AuNPs, with Cy3 positioned at the same set of distances **d-1, d-2,** and **d-3** (Figure 4e–g and Figure S20). Maximum quenching was again observed at **d-1,** where the normalized fluorescence intensity was reduced from 1 to 0.40 (±0.10, *N* = 3). The fluorescence was quenched from 1 to 0.68 (±0.06, *N* = 3) at **d-2** and from 1 to 0.89 (±0.06, *N* = 3) at **d-3** (Figure 4h). The same distance-dependent trend (**d-1** > **d-2** > **d-3**) was observed, consistent with the *in situ* metallized system. Notably, the *in situ* generated gold nanoclusters exhibited quenching efficiencies comparable to those of commercially sourced AuNPs, further validating the robustness and spatial controllability of our homemade site-specific reduction strategy.

### Strand Displacement Reaction-Mediated Programmable Fluorescence Switching

Finally, we designed a dynamic DNA origami system in which the Cy3 fluorophore could be displaced from the metallized template to restore the quenched fluorescence. For this, an 8 nt toehold domain was incorporated into the **Cy3-DNA** strand, which was hybridized to the protruding capture strand on the origami. The addition of 120 nM of the invader strand (**C-DNA**) that was fully complementary to the **Cy3-DNA** displaced the fluorophore-modified strand from the DNA origami into solution via TMSDR, resulting in the fluorescence recovery of the metallized origami (Figure 5a). Spectroscopic analysis showed that the Cy3-modified control system (at **d-1**) exhibited an “ON” state with normalized intensity *I* = 1, which decreased to 0.55 (±0.04, *N* = 3) after *in situ* metallization (”OFF” state). Subsequent addition of **C-DNA** led to the displacement of the **Cy3-DNA** from the wireframe template and complete fluorescence recovery with *I* = 1.07 (±0.07, *N* = 3), as shown in Figure 5b and Figure S21. An analogous wireframe origami template modified with externally synthesized AuNPs and Cy3 positioned at **d-1** was prepared for comparison. In this system, the addition of **C-DNA** resulted in the displacement of the **Cy3-DNA** from the template, leading to fluorescence recovery (Figure 5c). Fluorescence spectroscopy showed that the Cy3-modified control system had fluorescence turned “ON” (*I* = 1), which was quenched to 0.55 (±0.14, *N* = 3; fluorescence “OFF”) after AuNP attachment. **C-DNA** hybridized with **Cy3-DNA** and displaced it from the origami system, thereby resulting in near complete fluorescence recovery (*I* = 0.99 ± 0.02, *N* = 3) via TMSDR (Figure 5d and Figure S22).

**Figure 5:**
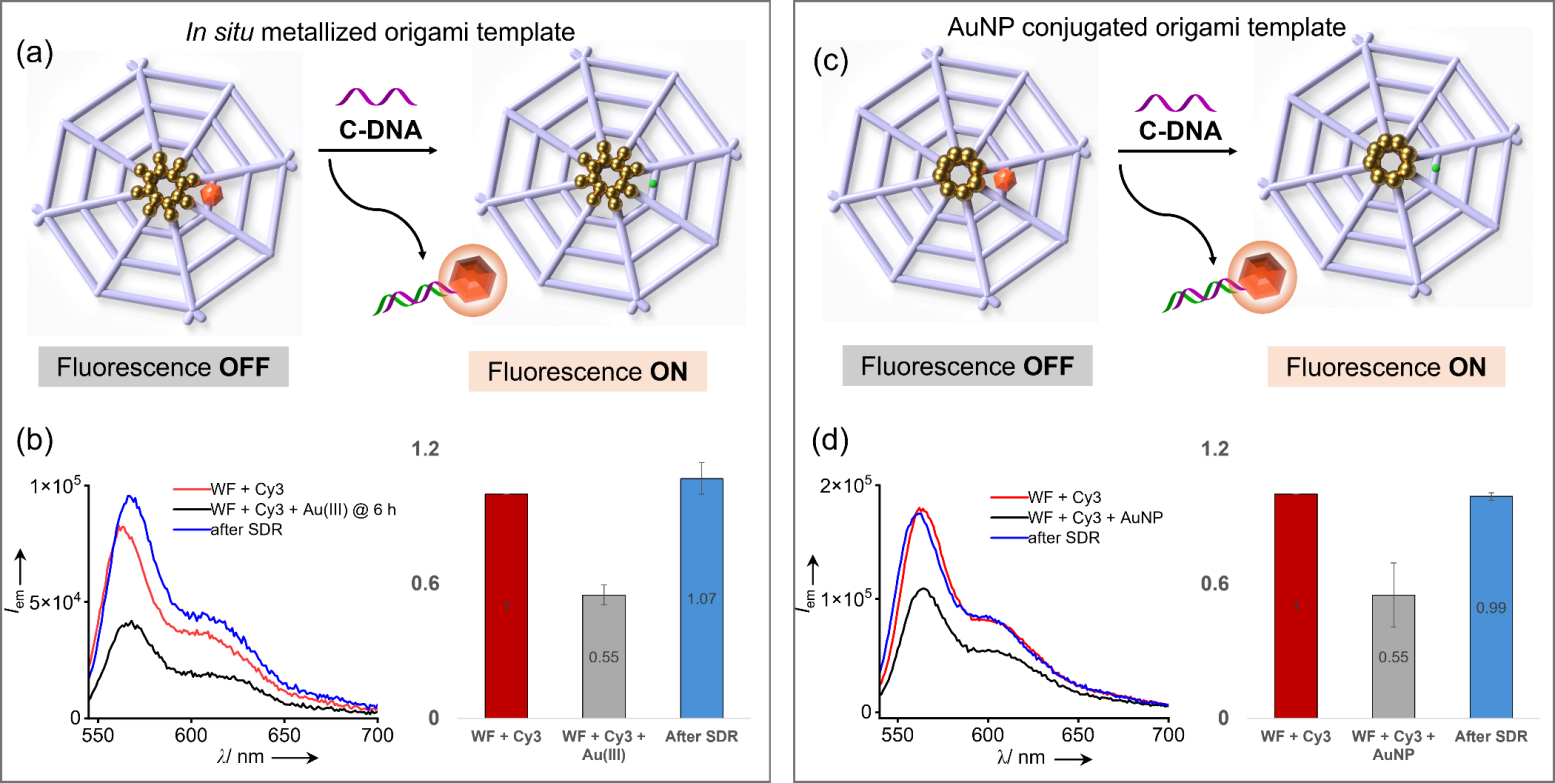
Strand displacement reaction (SDR)-mediated programmable fluorescence switch. (a) Schematic illustration showing the displacement of the **Cy3-DNA** from the *in situ* metallized wireframe origami template by the addition of an invader strand, **C-DNA**, to facilitate fluorescence recovery by SDR. (b) Emission spectrum showing the quenching of Cy3 fluorescence through *in situ* gold nanocluster formation and the subsequent complete fluorescence recovery after SDR (the normalized fluorescence intensity was quenched from 1 to 0.55 ± 0.04 after the metallization reaction, which was then fully recovered to 1.07 ± 0.07 after SDR). (c) Schematic illustration showing the displacement of the **Cy3-DNA** from the wireframe origami template conjugated with AuNPs by the addition of an invader strand, **C-DNA**, to facilitate fluorescence recovery by SDR. (d) Emission spectrum showing the quenching of Cy3 fluorescence by AuNPs and the subsequent complete fluorescence recovery after SDR (the normalized fluorescence intensity was quenched from 1 to 0.55 ± 0.14 after AuNP conjugation, which was then recovered to 0.99 ± 0.02 after SDR). *N* = 3 for all the experiments, and the normalized emission intensity is reported as mean ± SD.

## Conclusions

In this work, we first demonstrated the design and assembly of a spider-web-shaped wireframe DNA origami scaffold that serves as a template for *in situ* metallization, using our homemade **Iodo-DNA** conjugate as a site-specific nucleation initiator in HEPES buffer. This bottom-up nanofabrication approach relies on creating a localized, custom chemical environment that enables the C–I bond-mediated formation of gold nanoclusters with high yield at preprogrammed sites on the DNA origami template. We then leveraged this platform to demonstrate the time-dependent fluorescence quenching of a Cy3 fluorophore positioned proximally to the metallized sites. Systematic variation of the fluorophore–metal separation revealed that quenching efficiency is maximized at minimal distances and decreases with increasing separation, consistent with a distance-dependent energy transfer mechanism. Comparison with a reference origami system, in which externally synthesized 5 nm AuNPs were attached to the same wireframe template, showed comparable fluorescence quenching efficiencies, confirming that the *in situ* metallization strategy provides functionality similar to conventional AuNP attachment methods. Finally, we implemented a programmable fluorescence switch using TMSDR to displace the fluorophore from the template and reversibly restore the fluorescence of the quenched metallized origami.

In summary, we report an iodination-mediated strategy for the *in situ* generation of uniform gold nanoclusters under exceptionally mild reaction conditions, making this method readily compatible with biological systems. Previous studies have used close-packed origami templates to grow high-density arrays of metal nanoparticles.^15,30,59^ Our wireframe system is distinct in that it has an 8-fold symmetry, which allows the capture probes to be arranged in a symmetric pattern to a desired density, making it an ideal template for the point-specific growth of controlled metal nanoclusters. Moreover, their stability under low-salt conditions makes them ideal candidates for biomedical applications in physiological environments.^60^ This *in situ* reduction method may be translated to other close-packed origami systems described above to effectively generate high-density nanocluster patterns for various nanoelectronic applications by tuning the probe density and experimental conditions. Compared with conventional dye-quencher systems, the plasmonic origami system enables nanometer-scale control over dye positioning relative to the metallic nanoparticles. The distinctive geometric feature of the 8-fold wireframe origami allows the arrangement of multiple Fluorophores around the center nanoparticle clusters, enabling multiplexed detection of distinct analytes within a single platform. Overall, this work establishes a versatile and chemically orthogonal strategy for bottom-up nanofabrication, expanding the functional scope of DNA origami for applications in biosensing and photonics.

## Materials and Methods

### Synthesis of **2-IB**

All reagent-grade chemicals were purchased from Oakwood Chemicals and MilliporeSigma, USA. TLC analysis was performed using aluminum plates coated with silica gel-60 F254. Column chromatography was carried out using 200–400 mesh silica gel. Deionized ultrapure water (18.2 MΩ.cm) was used for all experimental steps. A 500 MHz Bruker Avance spectrometer was used to record ^1^H and ^13^C NMR spectra using tetramethylsilane (TMS) as an internal standard. A Bruker Ultra fleXtreme MALDI-TOF mass spectrometer was used for MALDI-TOF analyses.

### Attachment of DNA to Gold Nanoparticles

A 5 nm sized, citrate-capped AuNP was purchased from Ted Pella. Thiol-DNA (5′-thiol-TTTTTT TTTTGATTGACAGC-3′) was purchased from Integrated DNA Technologies. For AuNP-DNA formation, a mixture of 60 nM of AuNPs, 30 μM of thiol-DNA, and 350 μM of sodium dodecyl sulfate was prepared in deionized ultrapure water (total reaction volume—300 μL). This mixture was shaken at 30 °C and 800 rpm for 1 h. Following this, 5 μL of 2 M NaCl was added to the mixture every hour for 6 h. The mixture was then shaken at 800 rpm overnight. This mixture was then centrifuged at 15,000 rpm and 4 °C for 1 h so that the AuNP-DNA formed a pellet at the bottom of the tube, and the supernatant was discarded. The pellet was then redissolved in 300 μL of ultrapure water. These centrifugation steps were repeated two more times. After the third round of centrifugation and discarding the supernatant, the pellet was dissolved in 25 μL of ultrapure water. The AuNP concentration and absorbance spectrum were obtained using a Thermo Fischer NanoDrop spectrophotometer (extinction coefficient of AuNPs: 11,000,000).

### Preparation of Wireframe DNA Origami

M13mp18 single-stranded DNA (7249 bases), purchased from New England Biolabs (Catalog # N4040S), was used as the scaffold for origami assembly. DNA staple strands were purchased from Integrated DNA Technologies at 100 μM concentration in a plate and used as received without further purification. Wireframe origami with capture strands for **Iodo-DNA** was annealed in 1 mM HEPES buffer containing 12.5 mM magnesium acetate. Origami with capture strands for AuNP conjugation was annealed in 1X TAE buffer containing 12.5 mM magnesium acetate (TAE/Mg^2+^).

The mixture containing the scaffold DNA (3 nM) and staple strands (6 nM) in a suitable buffer was annealed with an Eppendorf PCR thermocycler by heating to 95 °C, followed by slow cooling to 4 °C at a constant rate over a period of 12 h: 94–86 °C by 4 °C every 5 min, 85–70 °C by 1 °C every 5 min, 70–40 °C by 1 °C every 15 min, 40–25 °C by 1 °C every 10 min, then held at 4 °C.

#### **Iodo-DNA** Conjugation

96 nM of **Iodo-DNA**, which was complementary to the capture strands of the assembled origami, was added, and the mixture was heated to 35 °C and gradually cooled to 4 °C over a period of 8 h. The following annealing protocol was used: 35–26 °C by cooling at a rate of 1 °C every 15 min, 25–10 °C by cooling at a rate of 1 °C every 20 min, then held at 4 °C.

#### AuNP Conjugation

48 nM of AuNP-DNA, which was complementary to the capture strands of the assembled origami, was added, and the mixture was heated to 45 °C and gradually cooled to 15 °C over a period of 13 h. The following annealing protocol was used: 45–20 °C by cooling at a rate of 1 °C every 30 min, 18 °C for 10 min, 16 °C for 10 min, then held at 15 °C.

**Cy3-DNA** (5′-Cy3 CATCGGTGGCAGTACGTCTCGC-3′) was hybridized with the protruding complementary strand on the wireframe origami template by adding 6 nM of **Cy3-DNA** to unpurified origami and heating the mixture to 30 °C and gradually cooling it down to 10 °C at a rate of 2 °C every 10 min. The mixture was then held at 4 °C.

The design and strand information can be found in Figure S3 and Table S1.

### AFM Imaging and Gel Electrophoresis

AFM imaging was performed using a Dimension FastScan AFM (Bruker) in “ScanAsyst in Liquid” mode. A ScanAsyst-Fluid+ tip from Bruker was used for imaging.

#### Sample Preparation for AFM Imaging

10 μL of 1X TAE/Mg^2+^ buffer was added to a freshly cleaved mica (Ted Pella) surface to form a uniform layer. 8 μL of origami sample was then deposited uniformly on the buffer layer. The mixture was incubated at room temperature for 10 min to allow attachment to the mica surface. This was followed by rinsing with 70 μL of 1X TAE/Mg^2+^ buffer to remove the excess staple strands. The rinsing step was repeated thrice. Finally, 70 μL of 1X TAE/Mg^2+^ buffer and 3.5 μL of a 100 mM NiCl_2_ solution were added to the mica and then imaged immediately.

0.9% agarose gel in 1X TAE/Mg^2+^ buffer was used to characterize the origami assembly. The gel was placed in a room-temperature water bath and was subjected to 75 V for 2 h. Gel staining was done with GelRed dye, and a 1 kb DNA ladder (from IBI) was used for gel band analysis.

### Fluorescence Spectroscopy

Steady-state fluorescence spectroscopy was performed on a Horiba Jobin Yvon Fluorimeter equipped with a thermostat Peltier cell holder, using a quartz cuvette (50 μL volume, 10 mm path length). The fluorescence measurement was performed at an excitation wavelength of 520 nm and an emission range from 535 to 700 nm. The mean ± SD was calculated from three individual experiments.

## Supplementary Material



## Notes

Reaction schemes and synthesis procedure of **2-IB** and **Iodo-DNA** (Synthesis Schemes), UV–vis spectrum and buffer optimization for gold nanocluster formation, HPLC chromatogram and MALDI-TOF spectrum of **Iodo-DNA**, wireframe origami design, additional AFM images of wireframe origami modified with **Iodo-DNA**, additional AFM images and statistical analysis of *in situ* metallized origami, negative control AFM images, height distribution analysis of origami-templated gold nanoclusters, TEM images of unmetallized and metallized wireframe origami, AFM images showing gold growth at different capture probe densities, agarose gel electrophoresis showing **Iodo-DNA**-modified wireframe origami treated with HAuCl_4_, additional AFM images and statistical analysis of wireframe origami modified with 5 nm AuNPs, negative control emission spectra, additional emission spectra showing the quenching of Cy3 fluorescence of *in situ* metallized and AuNP-conjugated wireframe origami systems and subsequent fluorescence recovery (Figures S1–S22), and strand information for the wireframe origami design (Table S1) (PDF)
