# Supplementary material for: Site-Specific In Situ Metallization of Wireframe DNA Origami for Reversible Fluorescence Switching
Source: ACS Nano. 2026 May 11;20(20):14574–83. doi: 10.1021/acsnano.6c00622 (PMC13218039; doi:10.1021/acsnano.6c00622)
Supplement: Supplementary file 1 [file nn6c00622_si_001.pdf]

# Supplementary information

# for

# Site-Specific *In Situ* Metallization of Wireframe DNA Origami for Reversible Fluorescence Switching

Minu Saji†, Devanathan Perumal†, Tiffany R. Olivera, Henry Wisniewski, Shaden Salim, Fei Zhang*

Department of Chemistry, Rutgers University, Newark, NJ 07102, USA

*Address correspondence to this author at Tel: 973-353-5520; E-mail: fei.zhang@rutgers.edu

## Table of Contents

**Synthesis Schemes** .......... 2

**Supplementary Figures** .......... 5

**Supplementary Tables** .......... 21

## Synthesis Schemes

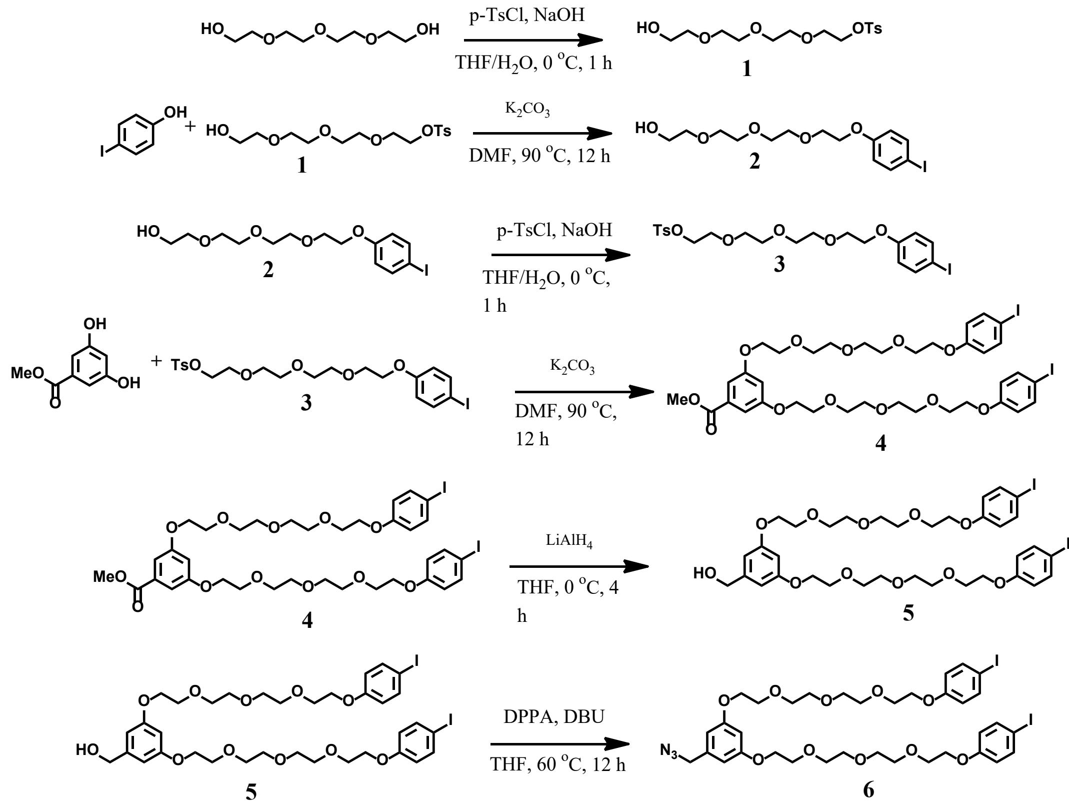


**Scheme 1.** Synthesis of **6 (2-IB).**

**Synthesis of 1**: In a 250 mL two necked round bottom flask, tetraethylene glycol (5.00 g, 25.7 mmol) was dissolved in 100 mL of THF. After the mixture was cooled to 0 °C, 15 mL of 50% aqueous NaOH (2.05 g, 51.5 mmol) was added. Then p-TsCl (4.40 g, 23.1 mmol) in THF was added slowly at 0 °C over a period of 1 h. After addition, the contents were allowed to warm up to

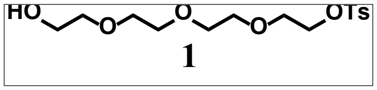


ambient temperature and stirred for another 1 h. Then the reaction mixture was extracted with DCM/water. The organic layers were collected, dried over anhydrous $Na_2SO_4$, concentrated in vacuum. The crude product was purified by silica gel chromatography using 70% PE/EA as eluent to give **1** as a colorless viscos liquid (72%); $^1$H NMR (500 MHz, $CDCl_3$), δ (ppm) = 7.73 (d, J = 5 Hz, 2H), 7.28 (d, J = 5 Hz, 2H), 4.10 (t, J = 5 Hz, 2H), 3.65-3.61 (m, 4H), 3.60-3.58 (m, 4H), 3.57-3.52 (m, 6H), 2.37 (s, 3H); $^{13}$C NMR (125 MHz, $CDCl_3$), δ (ppm) = 144.8, 133.0, 129.8, 127.9, 72.4, 70.7, 70.6, 70.5, 70.4, 70.3, 69.2, 68.7, 61.7, 61.7, 21.6.

**Synthesis of 2**: In a 100 mL two necked round bottom flask, 4-iodo benzaldehyde (1.9 g, 8.63 mmol) and oven dried $K_2CO_3$ (4.75 g, 34.4 mmol) was dissolved in 20 mL of dry DMF. After the mixture was heated to 45 °C for 30 min, compound **1** (3.0 g, 8.62 mmol) was added and then the contents were allowed to be heated up to 90 °C for 12 h. After completion of the reaction monitored by TLC, the DMF was

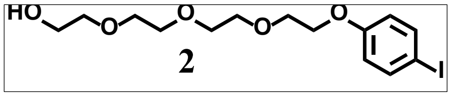

removed by vacuum concentration and then extracted with DCM/water. The organic layers were collected, dried over anhydrous $Na_2SO_4$, concentrated in vacuum. The crude product was purified by silica gel chromatography using 30% PE/EA as eluent to give **2** as a colorless oily liquid (79%). TLC (PE/EA): $R_f = 0.25$; $^1$H NMR (500 MHz, $CDCl_3$), δ (ppm) = 9.8 (s, 1H), 7.475 (d, J = 7 Hz, 2H), 6.633 (t, J = 7 Hz, 2H), 4.035 (t, J = 5 Hz, 2H), 3.780 (t, J = 5 Hz, 2H), 3.661-3.636 (m, 4H), 3.621-3.583 (m, 6H), 3.537 (t, J = 5 Hz, 2H); $^{13}$C NMR (125 MHz, $CDCl_3$), δ (ppm) = 158.6, 138.1, 117.0, 82.9, 72.4, 70.8, 70.6, 70.5, 70.3, 69.6, 67.5, 61.7.

**Synthesis of 3**: In a 50 mL two necked round bottom flask, compound **2** (2.0 g, 5.05 mmol) was dissolved in 25 mL of THF. After the mixture was cooled to 0 °C, 20 mL of 50% aqueous NaOH (1.2 g, 30 mmol) was added. Then p-TsCl (2.8 g, 14.6 mmol) in THF was added slowly at 0 ºC over a period of 1 h. After addition, the contents were allowed to warm up to ambient temperature and stirred for another 1 h. Then the reaction mixture was extracted with DCM/water. The organic layers were collected, dried over anhydrous $Na_2SO_4$, concentrated in vacuum. The crude product was purified by silica gel chromatography using 80% PE/EA as eluent to give **3** as an off-white oily liquid (72%). TLC (PE/EA): $R_f = 0.54$; $^1$H NMR (500 MHz, $CDCl_3$), δ (ppm) = 7.729 (t, J = 8.5 Hz, 2H), 7.473 t, J = 8.5 Hz, 2H), 7.269 (d, J = 8 Hz, 2H), 6.95 (d, J = 5 Hz, 2H), 6.628 (d, J = 5 Hz, 2H), 4.089 (t, J = 4.5 Hz, 2H), 4.023 (t, J = 4.5 Hz, 2H), 3.772 (t, J = 4.5 Hz, 2H), 3.628-3.600 (m, 4H), 3.578 (t, J = 3 Hz, 2H), 3.516 (s, 3H); $^{13}$C NMR (125 MHz, $CDCl_3$), δ (ppm) =138.19, 129.8, 127.9, 117.0, 70.7, 69.6, 69.2, 68.7, 67.5, 21.6.

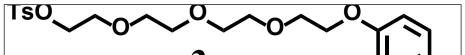


**3**

**Synthesis of 4**: In a 50 mL two necked round bottom flask, 3,5-dihydroxymethyl benzoyate (0.18 g, 1.07 mmol) and oven dried $K_2CO_3$ (0.88 g, 6.37 mmol) was dissolved in 10 mL of dry DMF. After the mixture was heated to 50 °C for 30 min, compound **3** (1.6 g, 2.90 mmol) was added and then the contents were allowed to be heated up to 90 °C for overnight. After completion of the reaction monitored by TLC, the DMF was removed by vacuum concentration and then extracted with DCM/water. The organic layers were collected, dried over anhydrous $Na_2SO_4$, concentrated in vacuum. The crude product was purified by silica gel chromatography using 30% PE/EA as eluent to give **4** as a colorless oil (81%). TLC (PE/EA): $R_f = 0.50$; $^1$H NMR (500 MHz, $CDCl_3$), δ (ppm) = 7.461 (d, J = 5 Hz, 4H), 6.620 (d, J = 5 Hz, 4H), 6.611 (s, 1H), 6.32 (d, J = 5 Hz, 1H), 4.063 (t, J = 4.5 Hz, 4H), 4.012 (t, J = 4.5 Hz, 4H), 3.781-3.754 (m, 8H), 3.653-3.637 (m, 8H), 3.617-3.600 (m, 8H); $^{13}$C NMR (125 MHz, $CDCl_3$), δ (ppm) = 159.7, 158.6, 138.1, 131.9, 117.0, 108.0, 10.6.9, 82.9, 70.8, 70.6, 69.6, 67.7, 67.5, 52.2.

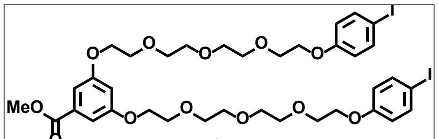


**4**

**Synthesis of 5**: $LiAlH_4$ (0.13 g, 3.4 mmol) was suspended in dry THF under $N_2$ atmosphere. Compound **4** (0.8 g, 0.86 mmol) in freshly distilled THF was added gradually and the reaction mixture was allowed to stir at room temperature for 2 h. Then the reaction mixture was quenched with ice cold saturated ammonium chloride and then filtered. The filtrate was concentrated in vacuum

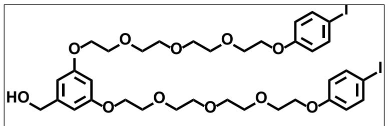


**5**

and purified using silica gel chromatography using 50% PE/EA as eluent to give **5** as a viscos liquid (95%). TLC (PE/EA): $R_f = 0.42$; $^1H$ NMR (500 MHz, $CDCl_3$), δ (ppm) = 7.211 (d, J = 7.5 Hz, 4H), 6.851 (d, J = 7.5 Hz, 4H), 6.457 (d, J = 2.5 Hz, 2H), 6.336 (s, 1H), 4.517 (s, 2H), 4.053 (d, J = 4.5 Hz, 4H), 4.030 (d, J = 4.5 Hz, 4H), 3.665-3.634 (m, 10H), 3.620-3.604 (m, 10H); $^{13}C$ NMR (125 MHz, DMSO-$d_6$), δ (ppm) = 160.1, 158.7, 143.3, 129.4, 120.8, 114.6, 105.4, 100.9, 70.8, 70.7, 70.6, 69.7, 67.5, 67.3, 65.2.

**Synthesis of 6**: In a 50 mL two necked round bottom flask, compound **5** (0.3 g, 0.33 mmol) was dissolved in dry THF under $N_2$ atmosphere. Then DPPA (0.2 g, 0.72 mmol) followed by DBU (0.1 g, 0.65 mmol) were added at room temperature and the reaction mixture was heated to 55 °C for overnight. The solvent was removed under vacuum and extracted with DCM/water. The organic layers were collected, dried over anhydrous $Na_2SO_4$, concentrated in vacuum. The crude mixture was purified by silica gel chromatography using 50% PE/EA as eluent to afford **6** as a brownish yellow oily liquid (90%). TLC (PE/EA): $R_f = 0.52$; $^1H$ NMR (500 MHz, $CDCl_3$), δ (ppm) = 7.212 (d, J = 7.5 Hz, 4H), 7.190 (s, 1H), 6.867 (d, J = 7.5 Hz, 4H), 6.385 (s, 1H), 6.380 (s, 1H), 4.059 (t, J = 4.5 Hz, 4H), 4.027 (t, J = 4.5 Hz, 4H), 3.794-3.754 (m, 8H), 3.670-3.639 (m, 10H), 3.626-3.608 (m, 10H); $^{13}C$ NMR (125 MHz, $CDCl_3$), δ (ppm) = 160.2, 158.7, 137.4, 129.6, 129.4, 120.8, 114.6, 106.9, 101.3, 70.8, 70.6, 69.7, 69.6, 67.5, 67.3, 54.8.

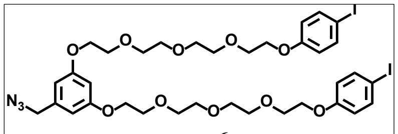

**6**

**Procedure for the synthesis of Iodo-DNA**

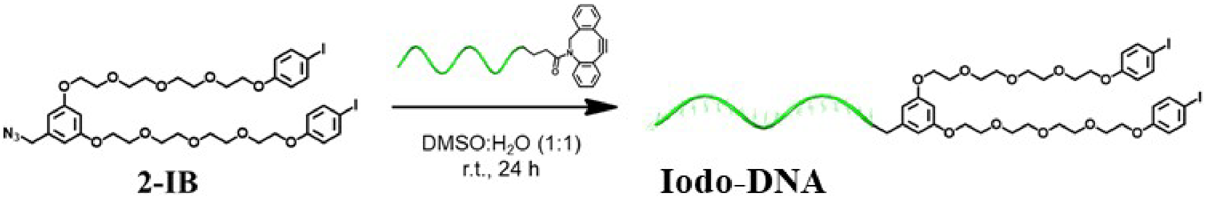

**2-IB** **Iodo-DNA**

5 μM of HPLC purified DBCO-modified DNA and 50 μM of **2-IB** was dissolved in 500 μL of 1:1 DMSO: H2O and the mixture was shaken at room temperature for 24 h. The reaction mixture was then vacuum concentrated using speed vacuum to remove the solvent completely, and the residue was extracted using water and ethyl acetate to remove unreacted **2-IB**. The ethyl acetate was carefully removed by pipetting, and the remaining aqueous layer was vacuum concentrated. This was followed by purification by 12% denaturing PAGE (buffer: 1 × Tris-Borate-EDTA, TBE, pH 8.3). The band containing **Iodo-DNA** conjugate was cut out and further characterized by HPLC and MALDI-TOF MS.

## Supplementary Figures

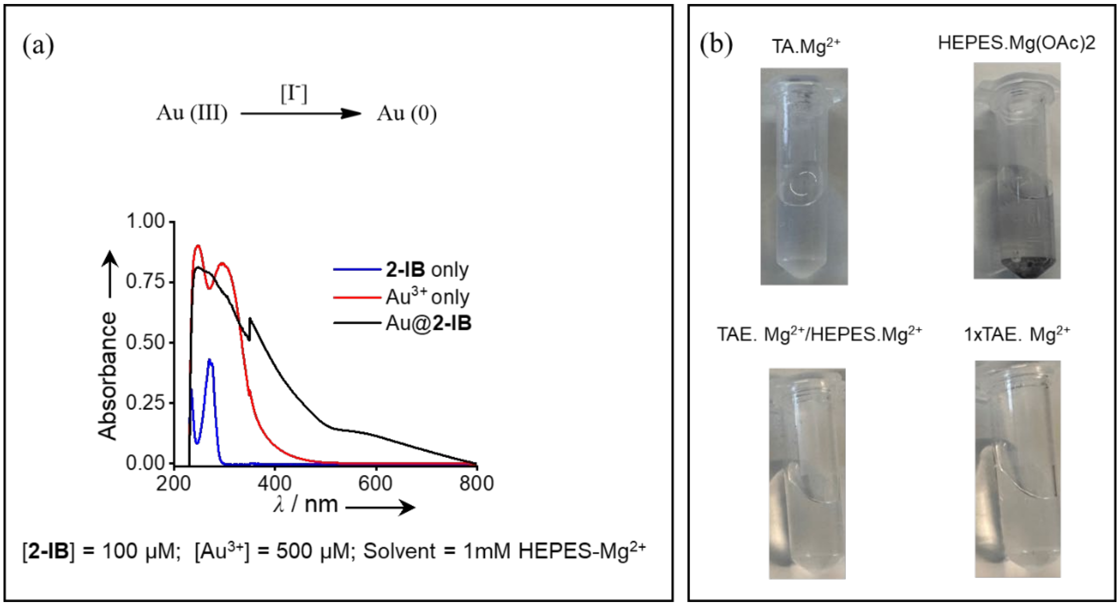


**Fig. S1**: (a) UV-Vis spectrum showing the appearance of plasmonic band (black line) when 100 µM of **2-IB** was treated with 500 µM of $HAuCl_4$ in 1mM HEPES-12.5 mM $Mg^{2+}$ buffer; the red and blue plots show the control experiments using the 500 µM of $HAuCl_4$ and 100 µM of **2-IB** respectively in the same buffer system. Plasmonic band is not visible in these control experiments, (b) **2-IB** mediated nanoparticle growth capability in various buffer systems like TA-$Mg^{2+}$, HEPES-$Mg^{2+}$, mixture of TAE-$Mg^{2+}$ and HEPES-$Mg^{2+}$, TAE-$Mg^{2+}$. Visible color change to purple was observed only in the case of HEPES-$Mg^{2+}$ buffer indicating successful reduction reaction in this condition.

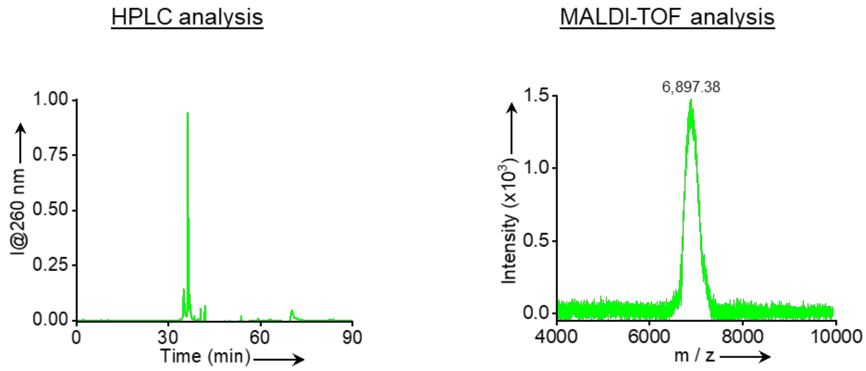


**Fig. S2**: HPLC chromatogram and MALDI-TOF spectrum of **Iodo-DNA** (calc. m/z = 6895.11)

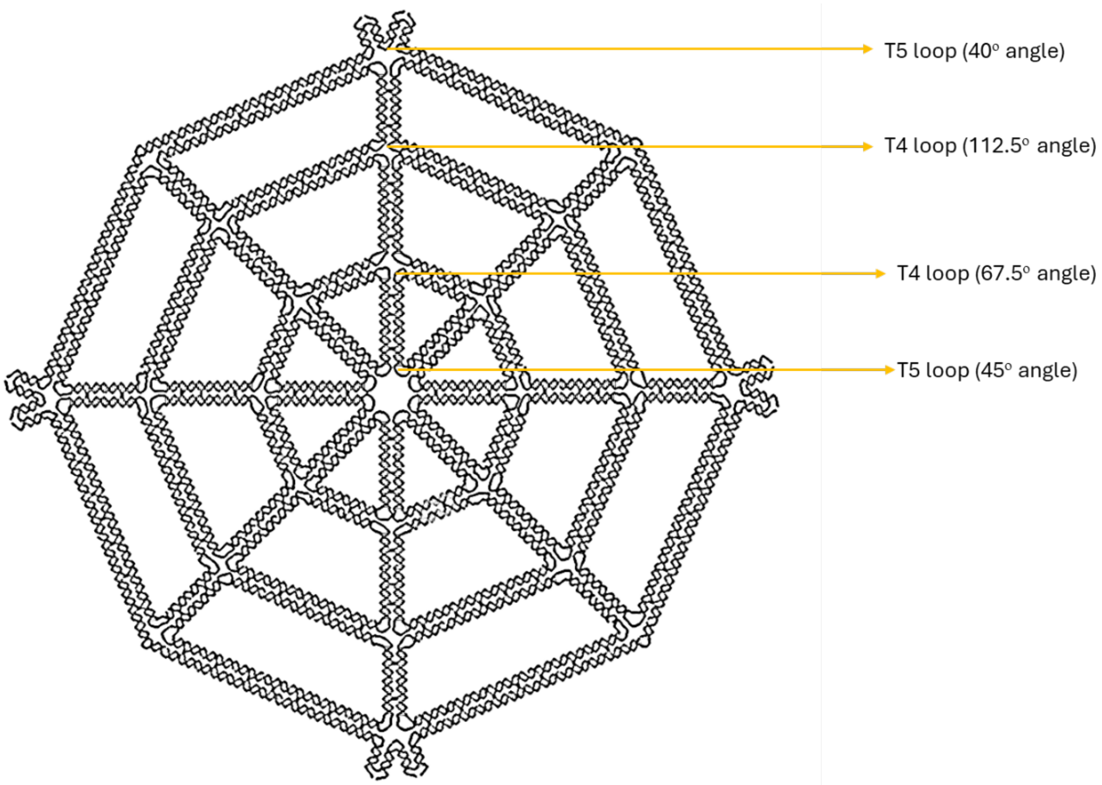


**Fig. S3**: Design of spider-web shaped wireframe DNA origami showing specific poly T loops at the junctions for angle control.

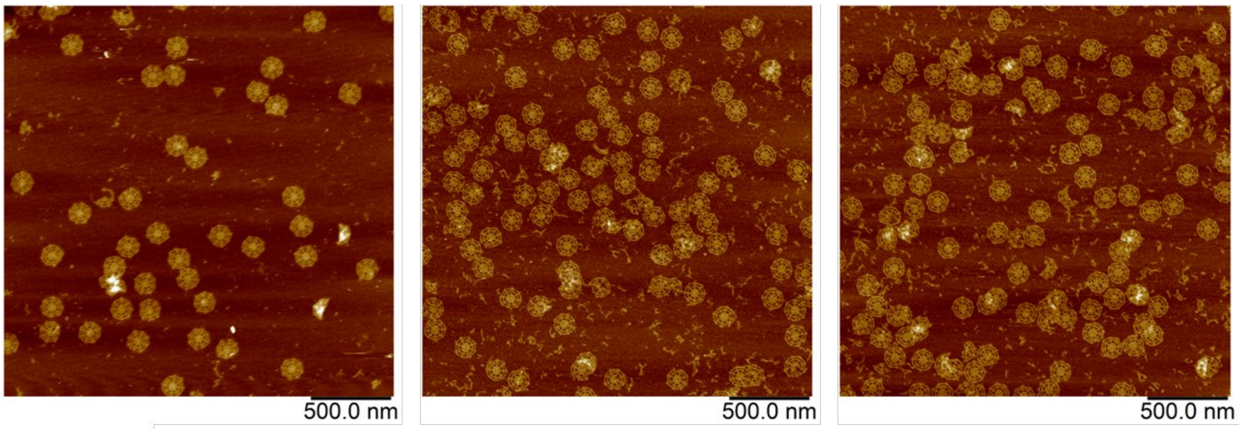


**Fig. S4**: Additional AFM images showing wireframe origami modified with **Iodo-DNA.**

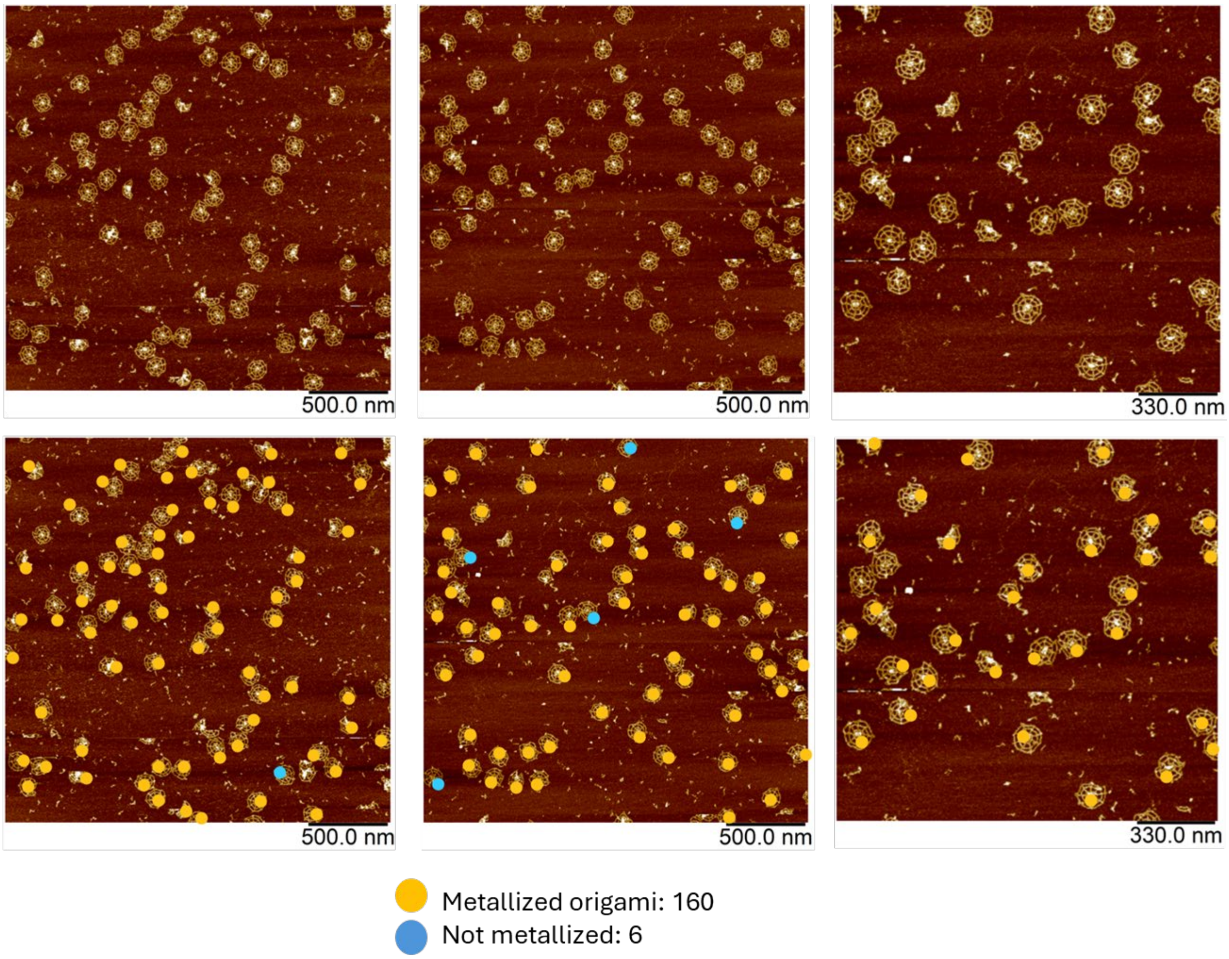


**Fig. S5**: Statistical analysis from AFM images showing the yield of successful metallization (N=166).

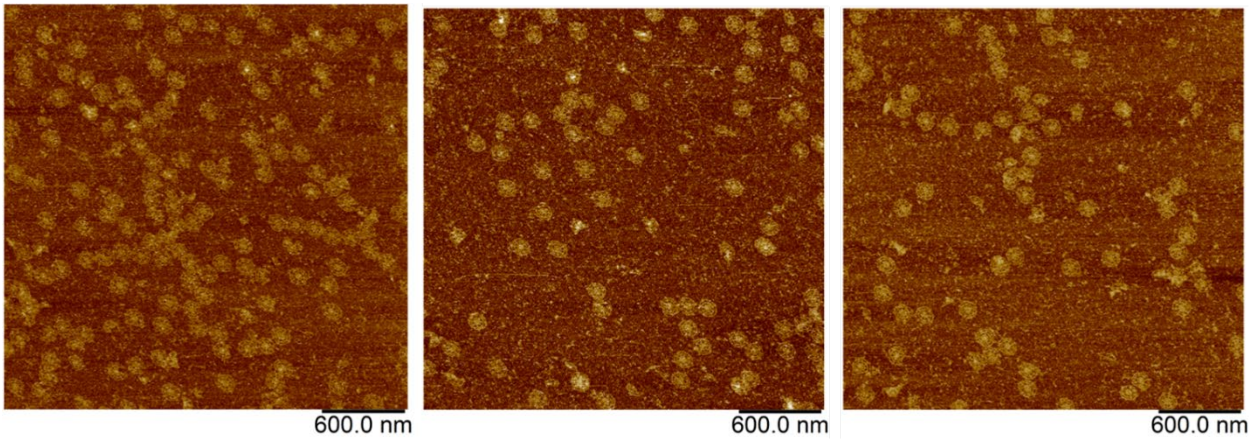


**Fig. S6**: Negative control experiment showing wireframe origami modified with double stranded DNA instead of **Iodo-DNA** and then treated with 50 µM $HAuCl_4$. No significant metallization is observed in this case, confirming that the metallization is mediated by **Iodo-DNA** chemical handle.

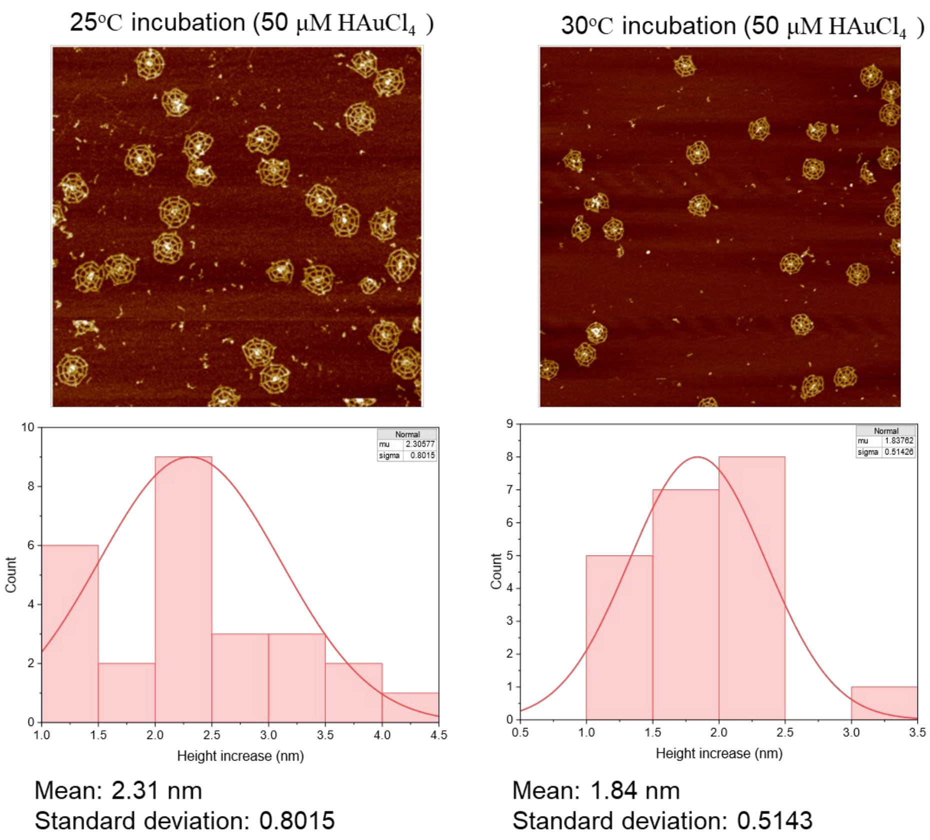


**Fig. S7**: Histograms showing the height distribution of gold metal growth on wireframe origami template with corresponding mean and standard deviations (for 25°C incubated sample, N=26; for 30 °C incubated sample, N=21).

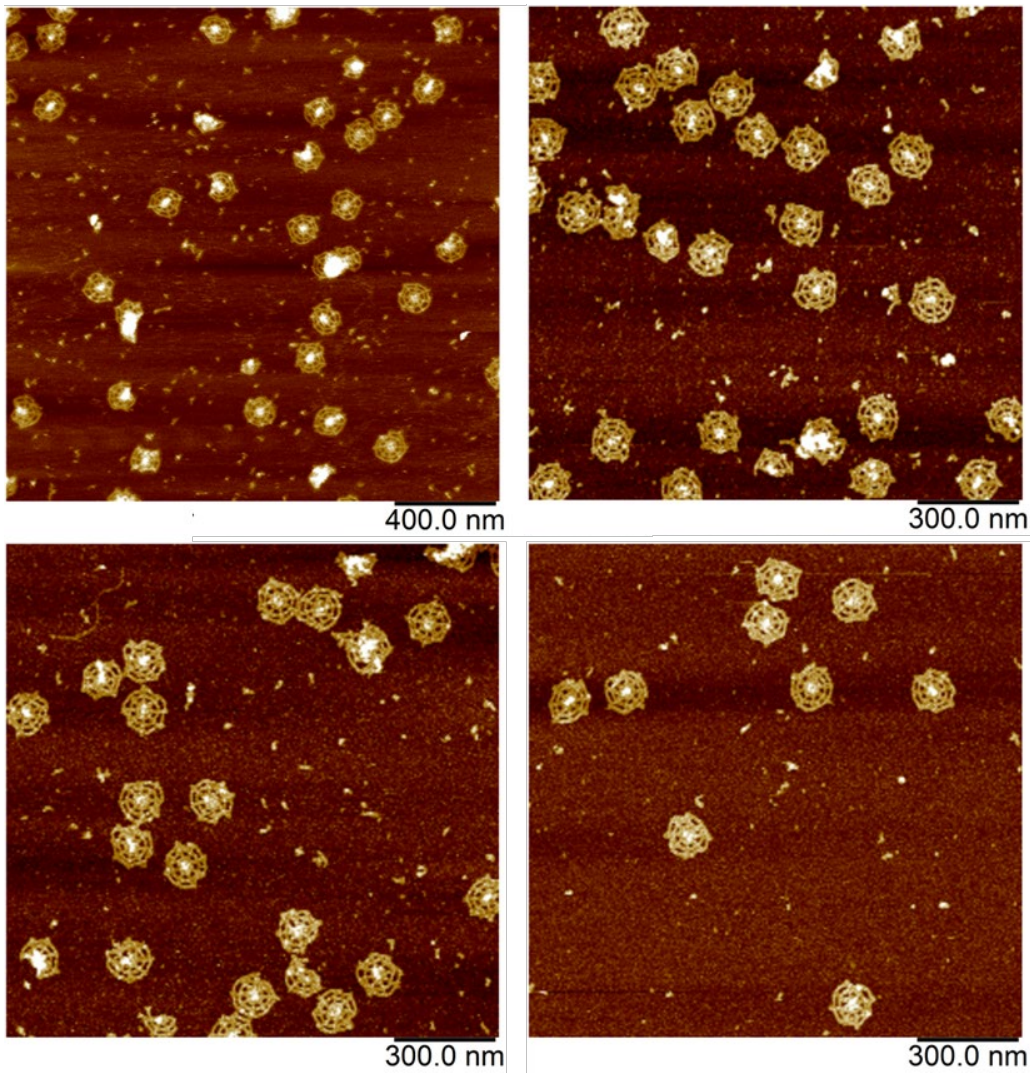


**Fig. S8**: Additional AFM images showing **Iodo-DNA** modified wireframe origami treated with 50 µM $HAuCl_4$ and incubated at 25°C.

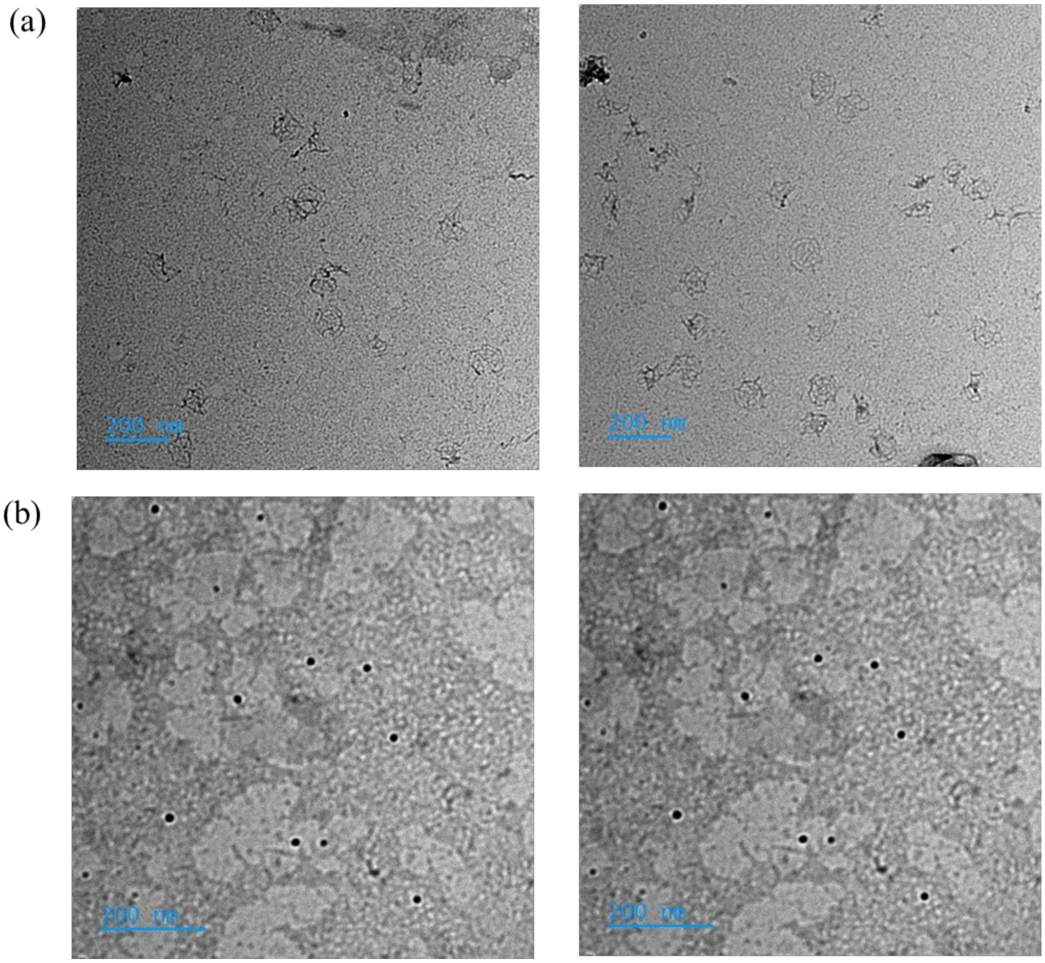


**Fig. S9**: TEM images showing (a) unmetallized wireframe origami template and (b) **Iodo-DNA** modified wireframe origami treated with 50 μM $HAuCl_4$ and incubated at 25ºC for *in situ* gold nanocluster formation. The origami templates are not visible in (b), but the nanoparticle formation is observed using TEM (Scale bar: 200 nm).

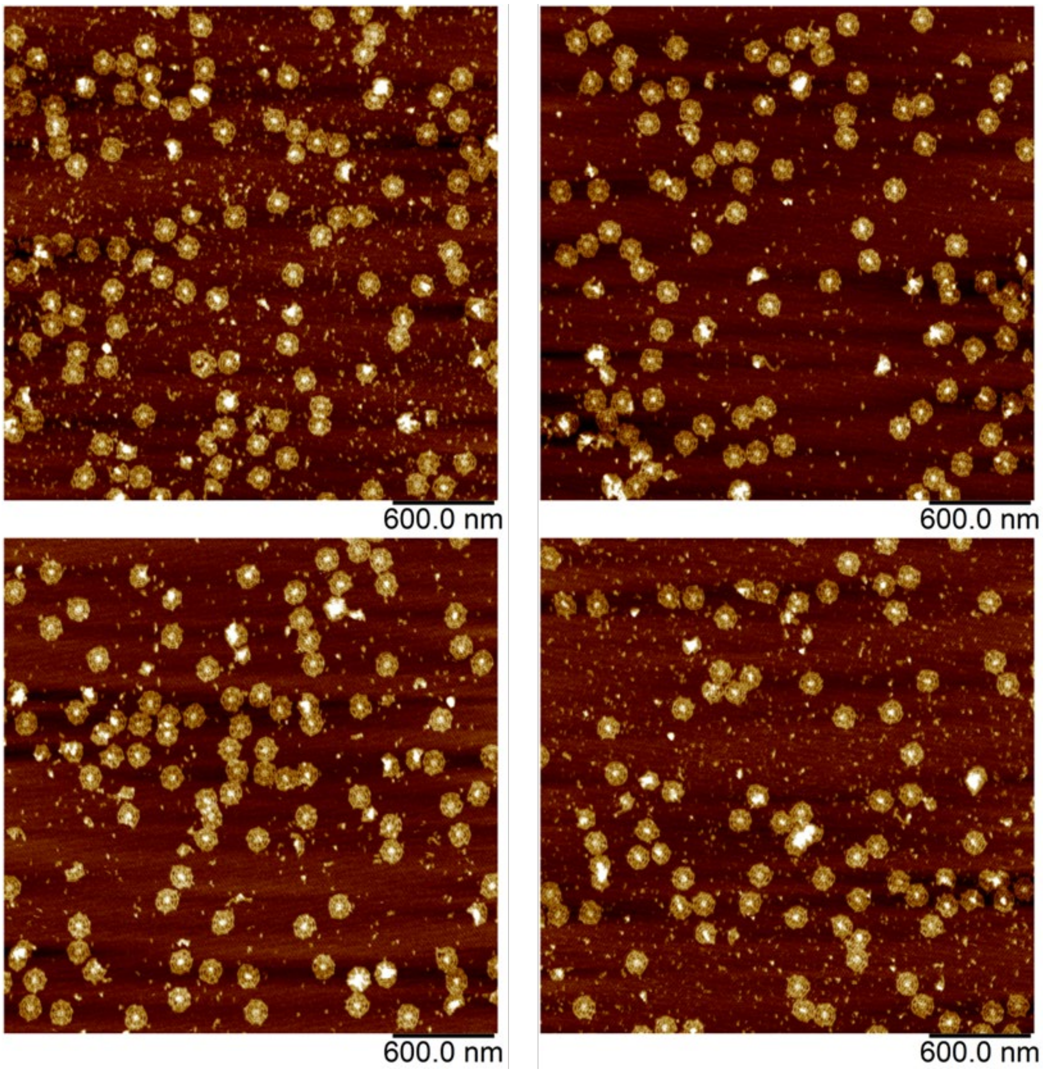


**Fig. S10**: AFM images showing the **Iodo-DNA** modified wireframe origami 24h after 50 µM $HAuCl_4$ addition (at 25ºC) showing stable nanoclusters localized to the central zone.

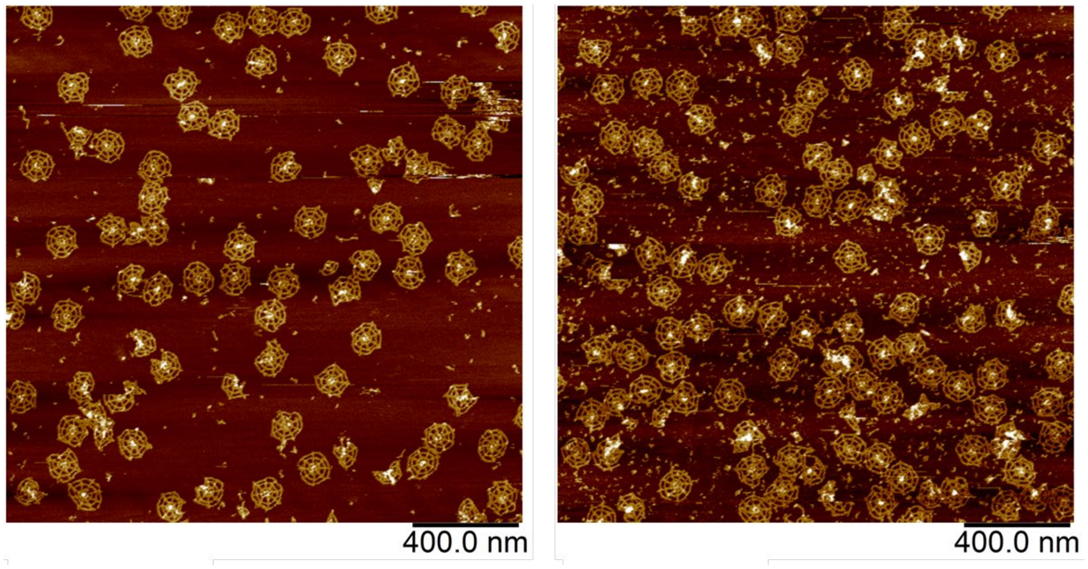


**Fig. S11**: Additional AFM images showing **Iodo-DNA** modified wireframe origami treated with 50 μM $HAuCl_4$ and incubated at 30°C.

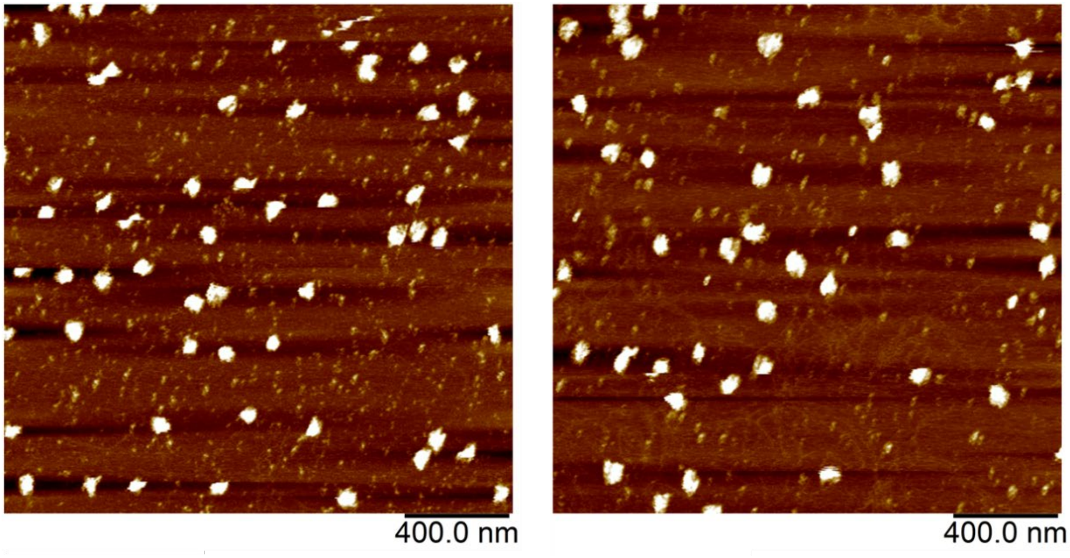


**Fig. S12**: Additional AFM images showing **Iodo-DNA** modified wireframe origami treated with 250 μM $HAuCl_4$ (10 min)**.**

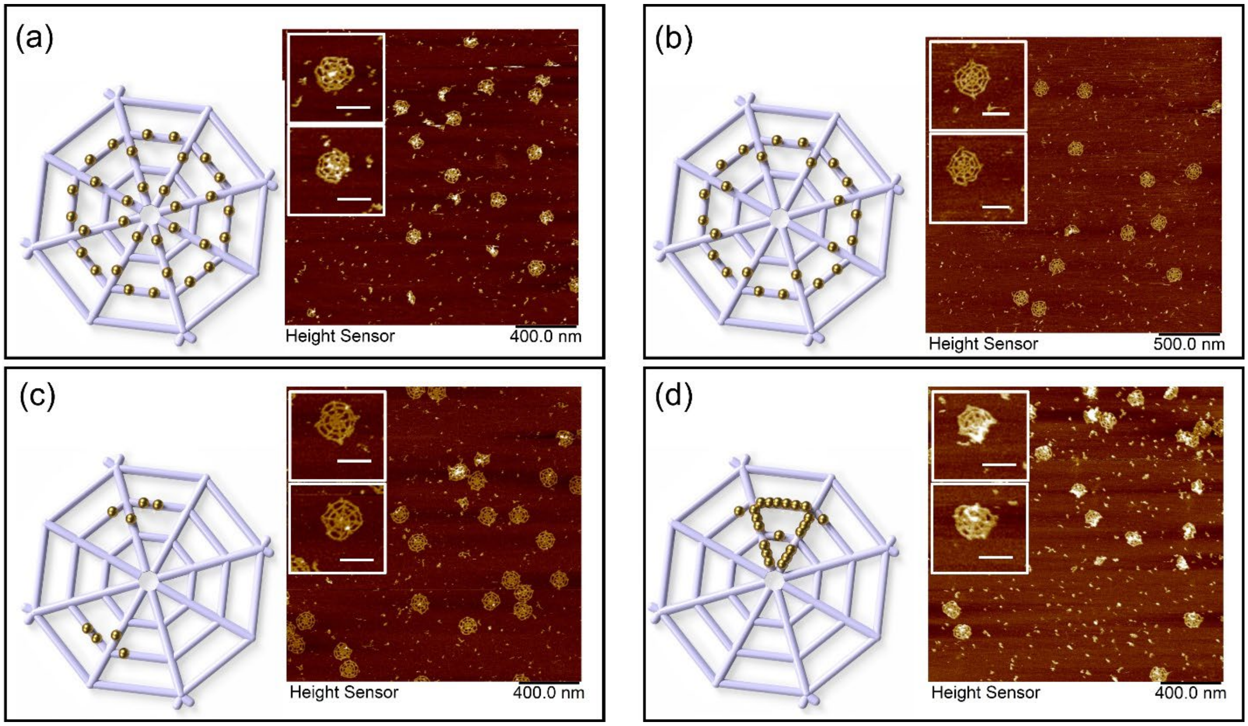


**Fig. S13**: Schematic design and corresponding AFM images showing wireframe origami design with (a) 32 capture probes distributed throughout the wireframe body showing metal growth predominantly in the innermost high density zone, (b) 24 capture probes modified at the outer zone of the origami showing no significant metal growth, (c) two sets of 4 clustered capture probes showing only ~21% yield of successfully metallized origami (in at least one of the two 4 probe clusters, N=28) and (d) triangular cluster of 21 capture probes with high density showing large particle growth (inset scale bar: 100 nm).

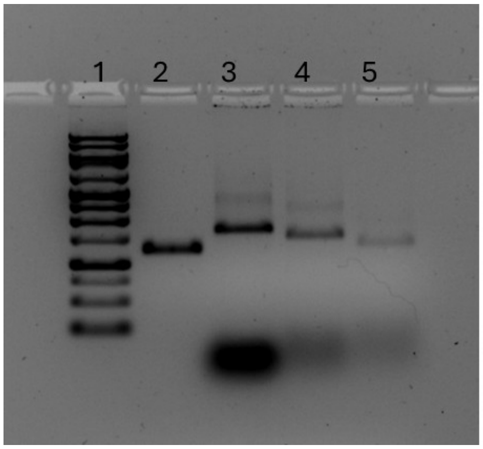


1. 1kb DNA ladder
2. M13 scaffold DNA
3. Wireframe origami-**Iodo-DNA** (control)
4. Wireframe origami- **Iodo-DNA-** 50 μM $HAuCl_4$
5. Wireframe origami- **Iodo-DNA-** 250 μM $HAuCl_4$

**Fig. S14**: Agarose gel electrophoresis (0.9 %) showing the treatment of **Iodo-DNA** modified wireframe origami with 50 μM $HAuCl_4$ and 250 μM $HAuCl_4$. The metallized wireframe origami shows a downward band shift due to the slight change in origami conformation due to shrinking

at the metallization site (the single-layer, mesh-like wireframe structure responds to the forces exerted due to the metal deposition, thereby resulting in a slight conformation change).

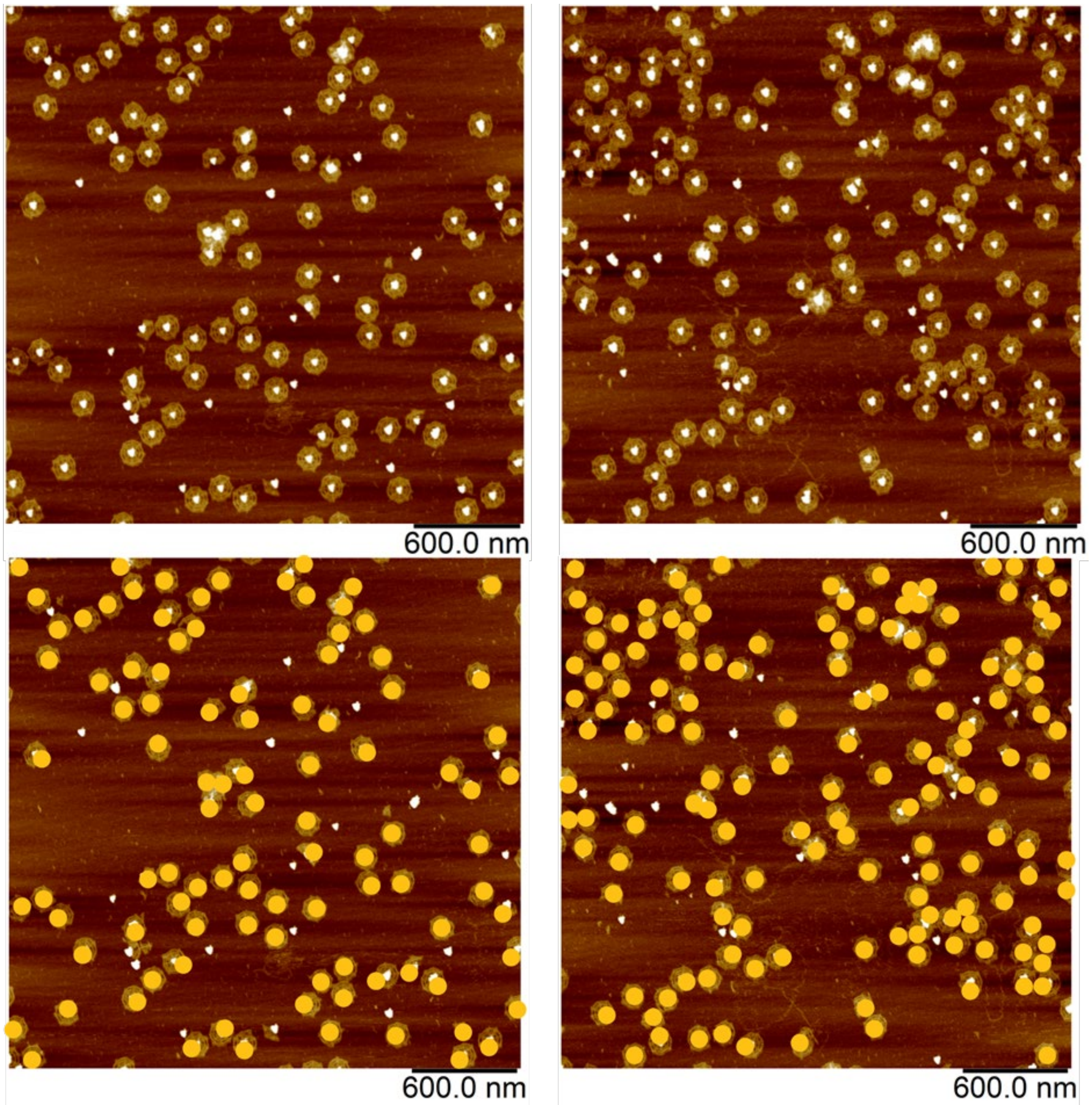


**Fig. S15**: Statistical analysis from AFM images showing the yield of successful AuNP conjugation to origami template (N=245). All the origami counted showed AuNP conjugation.

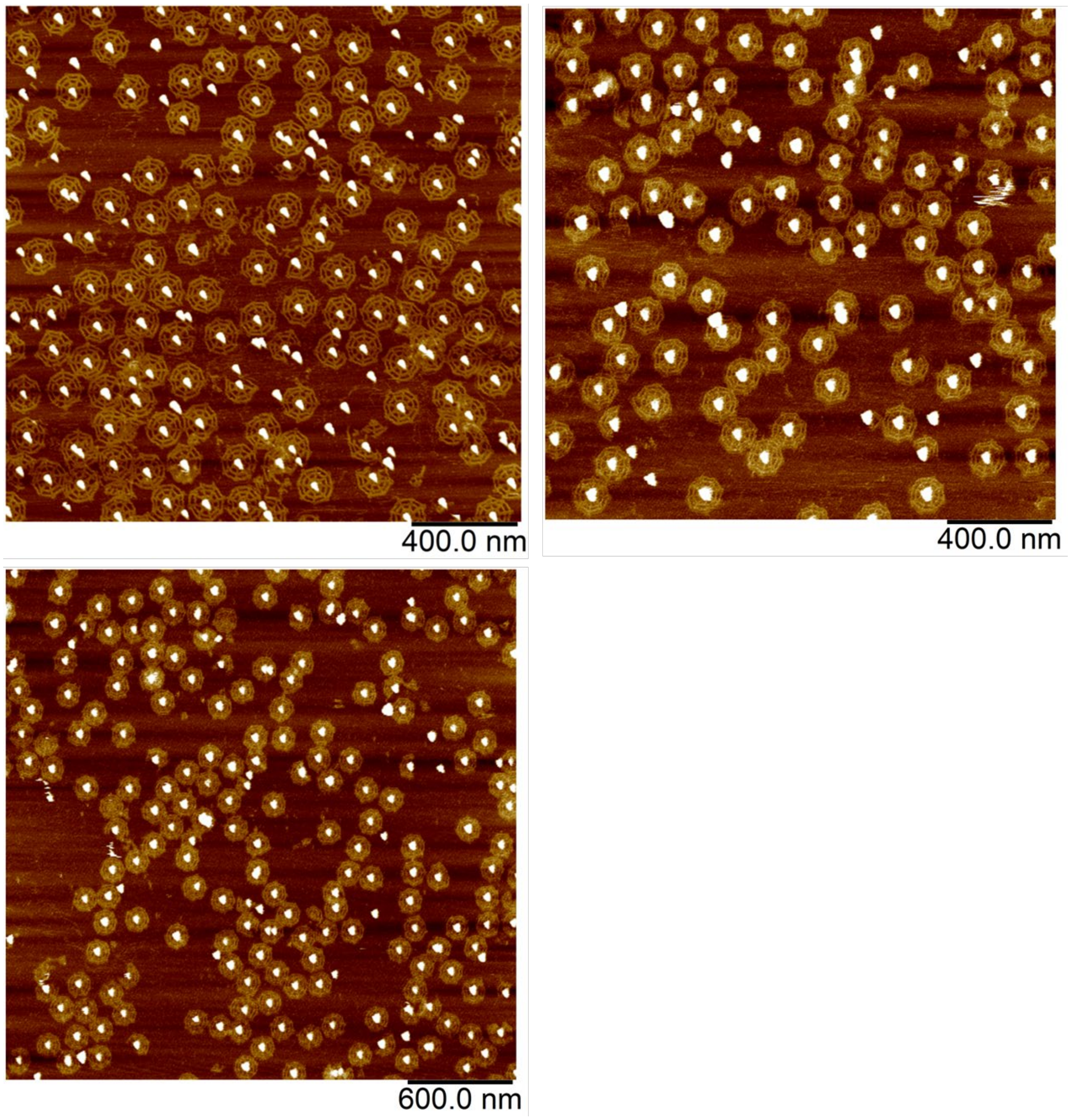


**Fig. S16**: Additional AFM images showing wireframe origami conjugated to 5 nm AuNP.

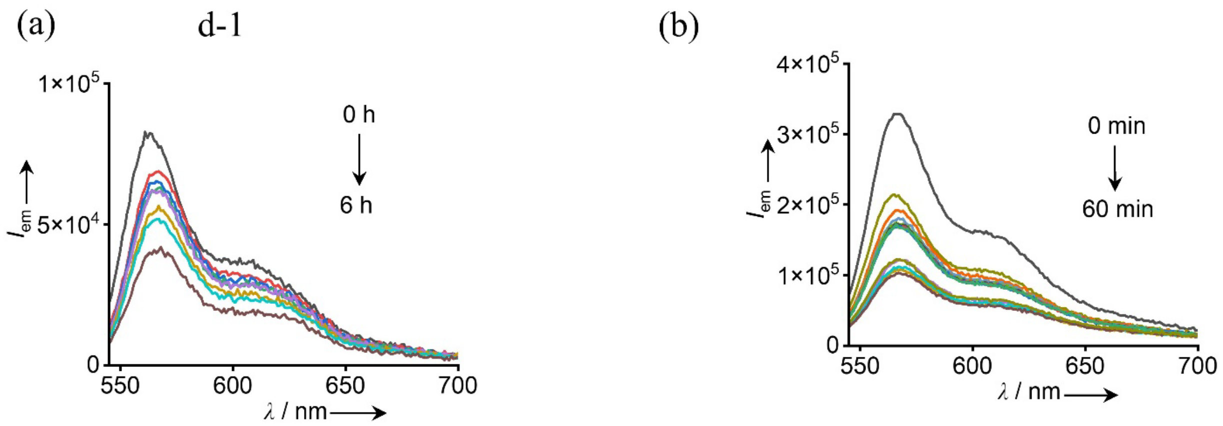


**Fig. S17**: Additional emission spectra showing time dependent quenching of Cy3 fluorescence when fluorophore is placed at distance **d-1** after treatment of **Iodo-DNA** modified wireframe origami with (a) 50 μM $HAuCl_4$ and (b) 250 μM $HAuCl_4$

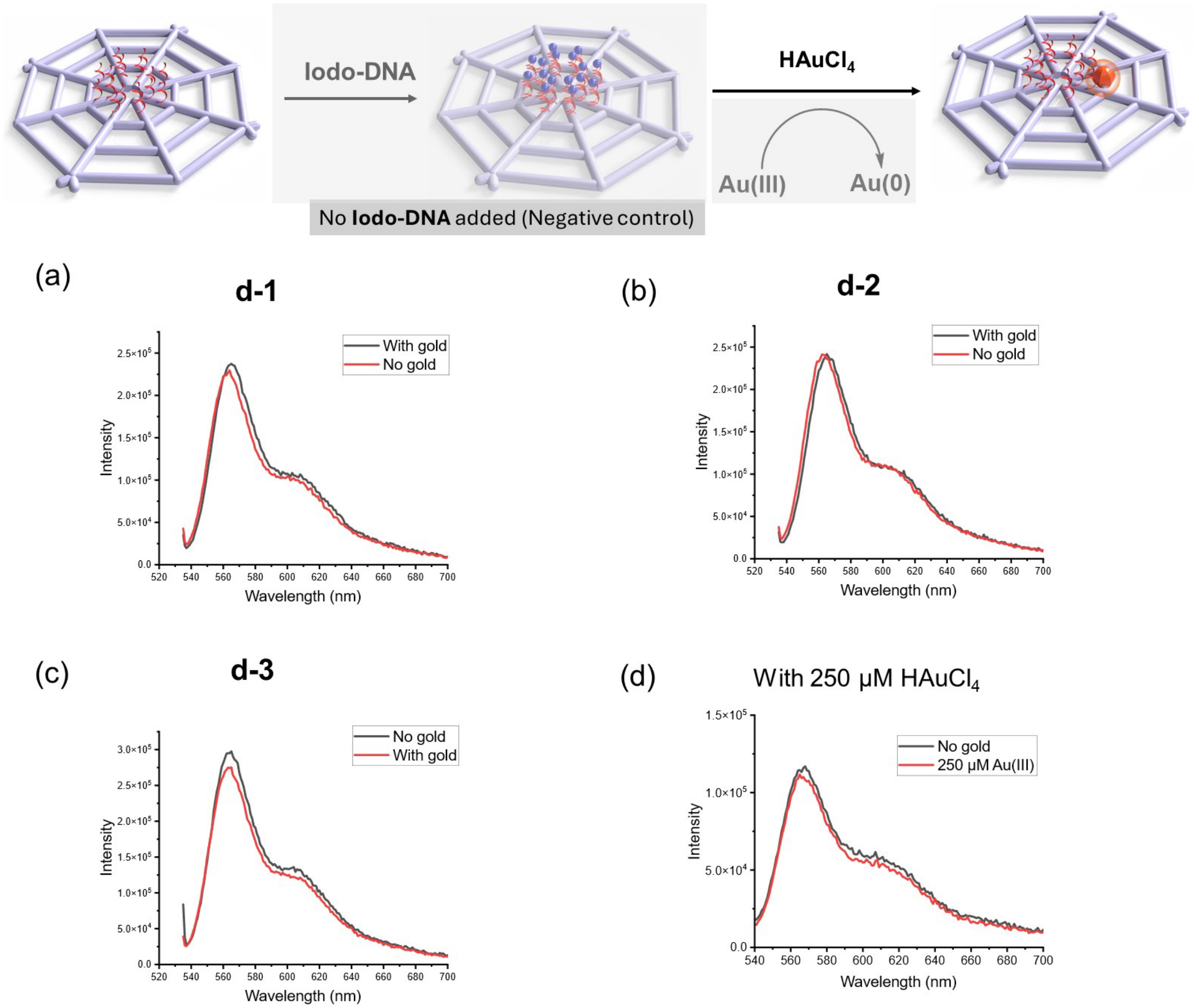


**Fig. S18**: Negative control emission experiments performed on Wireframe origami in 1 mM HEPES buffer without adding the **Iodo-DNA**. Wireframe origami modified with Cy3 at (a) **d-1**, (b) **d-2**, (c) **d-3**, treated with 50 µM HAuCl4 for 6h and (d) at distance **d-1** with 250 µM HAuCl4 for 1h. No significant quenching is observed when the negative control samples are excited at 520 nm.

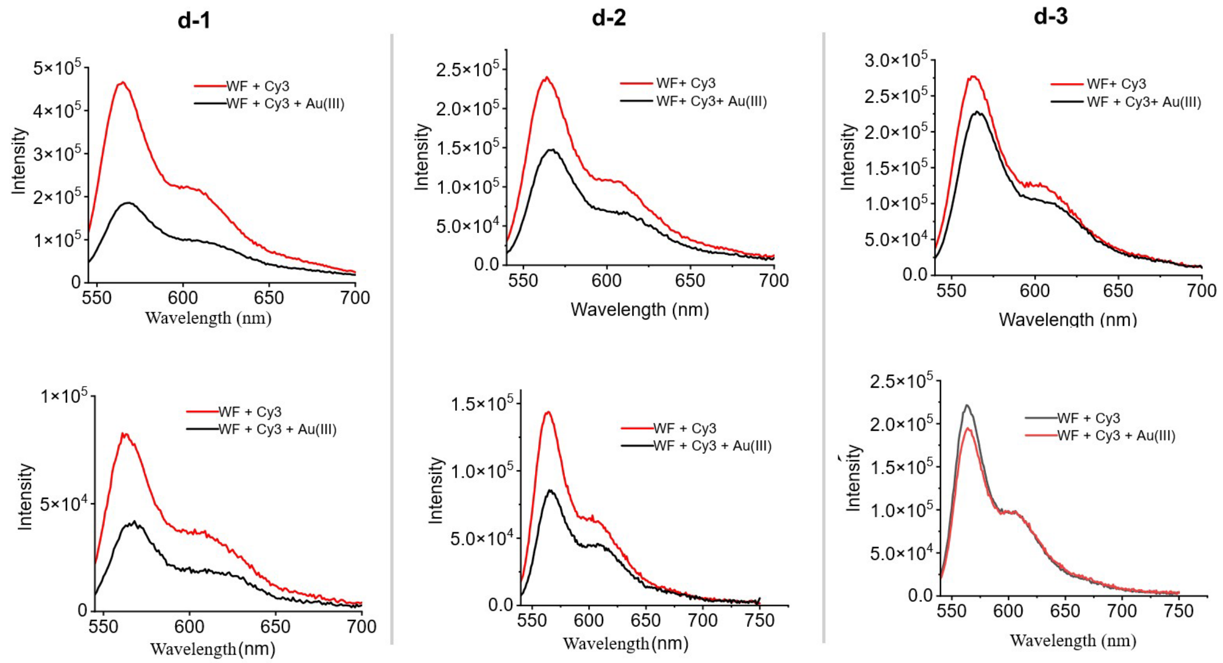


**Fig. S19**: Replicate emission spectra showing the quenching of Cy3 fluorescence when fluorophores are placed at distances **d-1, d-2** and **d-3** respectively after **Iodo-DNA** mediated *in situ* metallization**.** The quenching efficiency decreases with increase in separation.

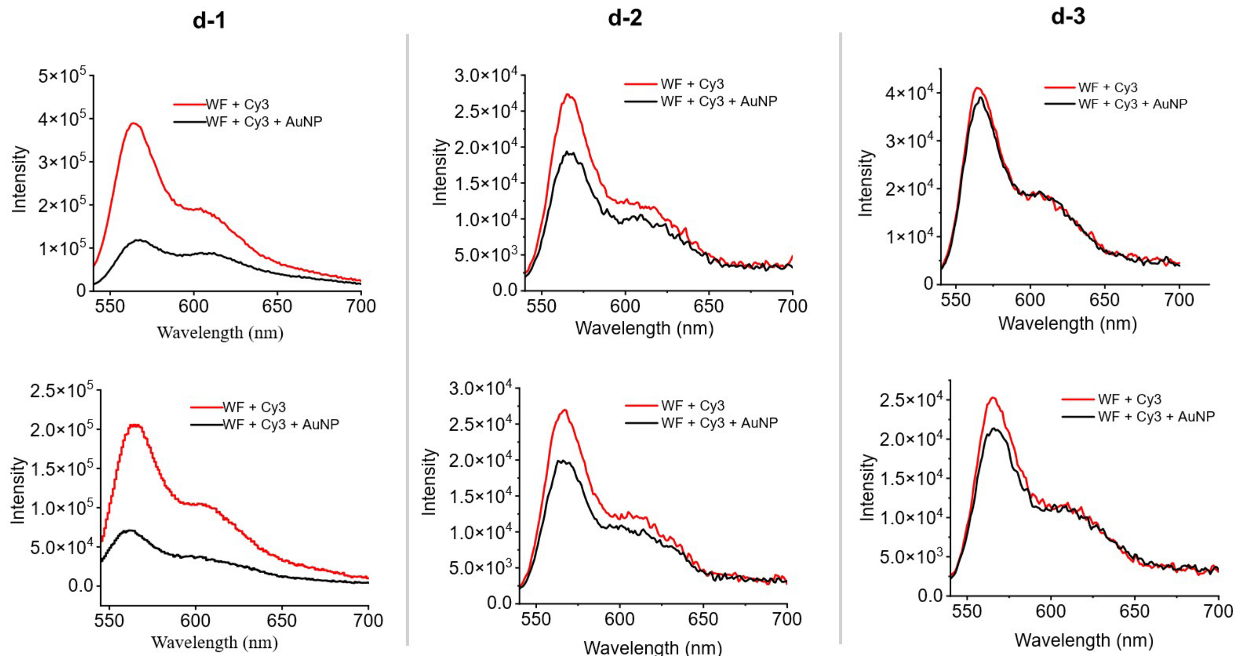


**Fig. S20**: Replicate emission spectra showing the quenching of Cy3 fluorescence when fluorophores are placed at distances **d-1, d-2** and **d-3** respectively after AuNP conjugation. The quenching efficiency decreases with increase in separation.

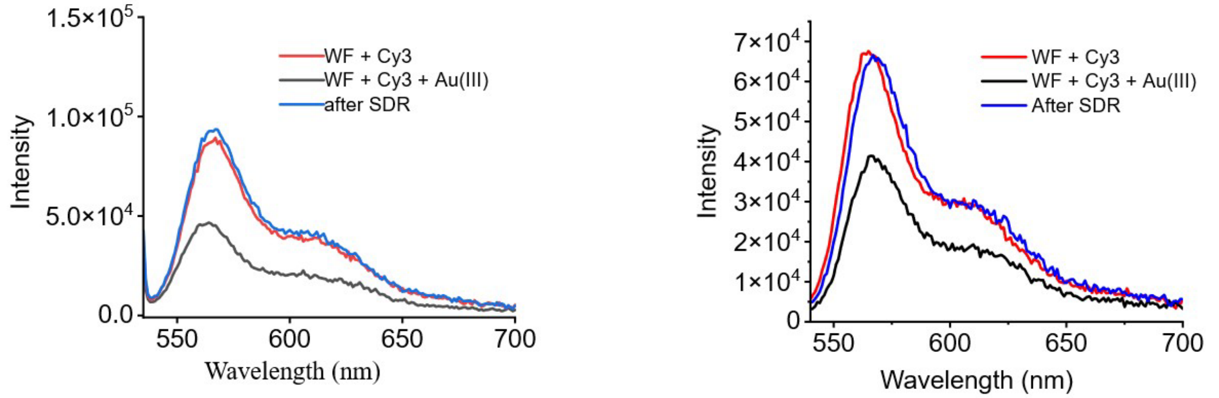


**Fig. S21**: Replicate emission spectra showing the quenching of Cy3 fluorescence by **Iodo-DNA** induced *in situ* metallization and subsequent fluorescence recovery after the addition of **C-DNA**

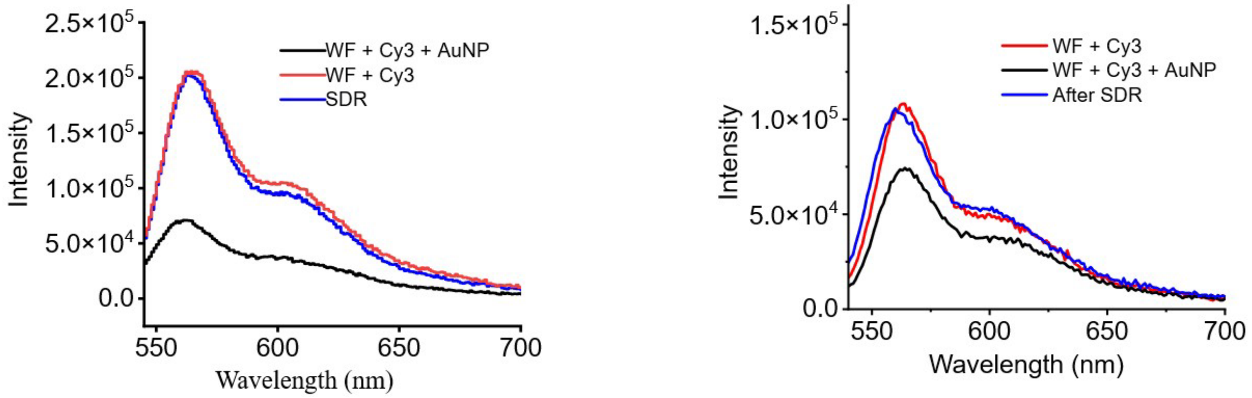


**Fig. S22**: Replicate emission spectra showing the quenching of Cy3 fluorescence by AuNP conjugation and subsequent fluorescence recovery after the addition of **C-DNA**

## Supplementary Tables

**Table S1:** Strand information for the wireframe origami design. Regions of the staple strands complementary to **Iodo-DNA** are marked in red.

| | |
|---|---|
| 2 | TGAGAAAGGCCGTAGTAGCATTAGCCTTTATTTTCAAAAGGG |
| 3 | GTAGGTAAAGATCAACGCAAGGATAAAAATTTTGAGTAATGT |
| 4 | GCAATGCCTTAGAACCCTCATATAAGGGGGAT |
| 5 | GTGCTGCATACGCCAGCTGGCGAATTTTAAAT |
| 6 | CTCTTCGCTATAGGCGATTAAGTTGGGTAACGCGGTGCGGGC |
| 7 | GGAAGGGCGATCCAGGGTTTTCCCAGTCACGAGCAACTGTTG |
| 8 | GGCTGCCGTTGTAAAATTTTCGACGGCCAGTGCCAATTTTT |
| 9 | AATTGTTTTCGTTGCGCTCAGCCATTCGCCTTTTATTCA |
| 10 | TTTTTGCTTGCATGCCTGCATTTTTGGTCGACTCTAGAGGATTTTT |
| 11 | TTTTTTCCCCGGGTACCGAGTTTTCTCGAATTCGTACATT |
| 12 | ATAGCTGTTTCGCCTAATGAGTGAGCTAACTCAATCATGGTC |
| 13 | TTGTTATCCGCATAAAGTGTAAAGCCTGGGGTCTGTGTGAAA |
| 14 | CACGTATACATACGAGCCGGAAGCTCACAATT |
| 15 | CCACACAAACGTGCTTTCCTCGTTGACGAG |
| 16 | CGGGAGCTAAGGGCGCGTACTATGGTTGCTTTAGAATCAGAG |
| 17 | GCGCCGCTACAACAGGAGGCCGATTAAAGGGATCCGCGCTTAAT |
| 18 | TAACTTTTCACCACACCCGTTTAGACAGGAACGGTACGCCATCCTGAGAAG |
| 19 | TGTTTTTATAACTTGCTGGTATTTTATATCCAGAACTGCGCG |
| 20 | AAACTATCGGCTCAGTGAGGCCACCGAGTAAAAGAAGAACTC |
| 21 | CTTGCCTGAGTAGAGTCTGTCCATCACGCAAAAATAACATCA |
| 22 | TGATTAGTTTAACCGTTGTAGCAAAACCACCA |
| 23 | GAAGGAGCTTTTGCGGAACAAAGATACTTCTT |
| 24 | TAACATTATCAGGAATTATCATCATATTCCTGAAAGTTTGAG |
| 25 | TATTAATTTTAATTATCAGATGATGGCAATTCGCCCGAACGT |
| 26 | CCTTTATCAATATAATTTTTCCTGATTGTTTGGATTTTTT |
| 27 | TACAATTTTAATCGCGCAGAACAACTCGTATTTTTTAAAT |
| 28 | TTTTTTATACTTCTGAATAATTTTTTGGAAGGGTTAGAACCTTTTT |
| 29 | TTTTTTACCATATCAAAATTTTTTATTTGCACGTACAAGT |
| 30 | AAAGAAATTGCTCGCCTGATTGCTTTGAATACAAACAGAAAT |
| 31 | AGGTTTAACGTTCGGGAGAAACAATAACGGATGTAGATTTTC |
| 32 | CTTTTACACAGATGAATATACAGTATAAAGTA |
| 33 | CCGACAAACCAGTAATAAGAGAATAACAGTAC |
| 34 | CATTTTCGAGAGGTAAAGTAATTCTGTCCAGTTAGGCAGAGG |
| 35 | TAAACAACATGTTAACAACGCCAACATGTAATACGACGACAA |
| 36 | TTGAGTTTTAATCGCCATATTTCAGCTAATGCAGAACGCGGTTTATCAAC |
| 37 | AATAGATAAGTTCGAGAACAATTTTGCAAGCCGTTTCTTAA |
| 38 | AAAATAATATTTAAACCAAGTACCGCACTCACCTGAACAAGA |
| 39 | TTACGAGCATTTATCATTCCAAGAACGGGTACCCATCCTAAT |

| | |
|---|---|
| 40 | GTCTTTCCGTAGAAACCAATCAATCATAAAGG |
| 41 | TGGCAACAACGTAGAAAATACATAAATCGGCT |
| 42 | TATGTTAGCAATATAAAAGAAACGCAAAGACATATTACGCAG |
| 43 | TTAAGACTCCTCCACGGAATAAGTTTATTTTGACTGGCATGA |
| 44 | CACCATTTTATGAAACCATCAATAACGGAATTTTTACCCAAAAGATCACA |
| 45 | ATCAATTTTTAGAAAATTCATATGGTTTTT |
| 46 | TTTTTTTTACCAGCGCCAAATTTTTGACAAAAGGGCGACATTTTTT |
| 47 | TTTTTTCAACCGATTGAGGGTTTTAGGGAAGGTAAAACGT |
| 48 | AAATTATTCATATTACCATTAGCAAGGCCGGAATATTGACGG |
| 49 | TTATCACCGTCAGCAAAATCACCAGTAGCACCTAAAGGTGAA |
| 50 | TTAGAGCCACCGACTTGAGCCATTTTAGTACC |
| 51 | GCCACCCTTCACCGTACTCAGGAGGTTGGGAA |
| 52 | ACCCTCAGAACGTATAGCCCGGAATAGGTGTACAGAACCGCC |
| 53 | AGAGCCACCACCCGTCGAGAGGGTTGATATAACGCCACCCTC |
| 54 | AAGTGCCTCATTTTCAGGGATAGCACCAATAGGAA |
| 55 | CCCATGTACCGATTTTCTGTATTTTTGGGATTTTGCTCAGTACCAGTTTTGCGGAT |
| 56 | TTCGTCACCATTTCCAGACGTTAGTAAATGATAACACTGAGT |
| 57 | AACGCCTGTAACGATCTAAAGTTTTGTCGTCGTACAAACTAC |
| 58 | TTAGCGTAGCATTCCACAGACAGCACAGACCA |
| 59 | GGCGCATAGACAGATGAACGGTGTCCTCATAG |
| 60 | CTTTGAAAGAGGGCTGGCTGACCTTCATCAAGAACTGACCAA |
| 61 | TAAGGGAACCGAGTAATCTTGACAAGAACCGGCGGTCAATCA |
| 62 | CGCAGAATATTCATTATTTTCCCAAATCAACGTAACTTTTT |
| 63 | TTTTTAAAGCTGCTCATTCATTTTTGTGAATAAGGCTTGCCTTTTT |
| 64 | TTTTTCTGACGAGAAACACCTTTTAGAACGAGTAGTAAAA |
| 65 | CGAACTTTTTAACGGAACAATTAGCCGGAATTTTCGAGG |
| 66 | TTGAGATGGTTGGGAAGAAAAATCTACGTTAATAAATTGGGC |
| 67 | TTTAATCATTGCATTATACCAGTCAGGACGTTTAATTTCAAC |
| 68 | AACTGGCTTGAATTACCTTATGCGTTAGTTTG |
| 69 | ACCATTAGCTGCGAACGAGTAGATATTTTAAG |
| 70 | ATTCCCAATTATACATTTCGCAAATGGTCAATATAACAGTTG |
| 71 | TTTCATTCCATAACCTGTTTAGCTATATTTTGTGTCTGGAAG |
| 72 | ATGCATTTTACTAAAGTACGCATTTGGGGCGCGAGCTGAAGTGGCATCAA |
| 73 | TTCTACTAATAGGAGACAGTCTTTTAAATCACCATCTAAAT |
| 74 | AAGAGAATGAGGGTAGCTATT |
| 75 | TTTGAGAGGTCTGGAGCAAAC |
| 76 | GCCTGAGAATCTACAAAGGCTATCCGACGACA |
| 77 | GTATCGGCTGCCAGTTTGAGGGGAAGGTCATT |
| 78 | CACTCCAGGGCGCATCGTAAC |
| 79 | CGTGCATCCTCAGGAAGATCG |
| 80 | AGATGCCAGCTTTCCGTTTTCACCGCTTCTGAACCTGTCGTTTTTCCAGC |
| 81 | CCAGGCAAAGCCTGCCCGCTTTCCAGTCGGGAGTGCCGGAAA |

| | |
|---|---|
| 82 | TGCATAGACGGGCAACTTTTAGCTGATTGCCAGGTCACGTTTTTTGGTGT |
| 83 | CACCAGTGTAATGAATCGGCC |
| 84 | AACGCGCGTGGTTTTTCTTTT |
| 85 | CGCCAGGGGGGAGAGGCGGTTTGCAGCCGGCG |
| 86 | AACGTGGCAGAGCTTGACGGGGAAGTATTGGG |
| 87 | CCCGATTTGAGAAAGGAAGGG |
| 88 | AAGAAAGCCCTAAAGGGAGCC |
| 89 | GGAACGAAAGGAGCGGTTTTCGCTAGGGCGCAACAGGAAAATTTTCGCT |
| 90 | AGCGGTCACGCAATATTACCGCCAGCCATTGCTGGCAAGTGT |
| 91 | CATGGATAATAAAAGGGTTTTACATTCTGGCCGTAAAGCACTTTTTAAATC |
| 92 | ACGACCAGAATACCTACATTT |
| 93 | TGACGCTCATTCACCAGTCAC |
| 94 | ATTGGCAGAATCGTCTGAAATGGAGCACTAAC |
| 95 | AACTAATATAAAATATCTTTAGGATTATTTAC |
| 96 | AGGTTATCGATTAGAGCCGTC |
| 97 | AATAGATAAAGGAATTGAGGA |
| 98 | GTGAGGTTGGCAAATTTTTCAACAGTTGAATACATTTGAGTTTTATTTAG |
| 99 | AAGTAACCTGAGCAATTTTAGAAGATGATTACATAAATCATTTTATATAT |
| 100 | CAAACAATTCGGGCGAATTATTCATTTCAATTTTAGACTTTA |
| 101 | GGAAACAGGAAACAAACATCA |
| 102 | AGAAAACATACCTTTTTTAAT |
| 103 | ATTTGAATAAATTAATTACATTTATAATTACT |
| 104 | AGAAAAAGATAAACACCGGAATCAACAATTTC |
| 105 | AAATAAGACCTGTTTAGTATC |
| 106 | ATATGCGTTAAATAAGGCGTT |
| 107 | TGTGATATACAAATTCTTTTTACCAGTATAATTACCGCGCCTTTTAATAG |
| 108 | CAAGCGTTTTGAAGCCTTTTTAAATCAAGATTGAAATACCTTTTGACCG |
| 109 | CAACAGTAGGGTTATTTTCATCGTAGGAATCAAGCCAACGCT |
| 110 | TGCGGGAGAAATCAGATATAG |
| 111 | AAGGCTTAGAACCTCCCGACT |
| 112 | GTTTTAGCTCCGGTATTCTAAGAAGCTATCTT |
| 113 | ACCGAAGCCAATGAAATAGCAATACGCGAGGC |
| 114 | GCAAGAAACCTTTTTAAGAAA |
| 115 | AGTAAGCACCCAATAATAAGA |
| 116 | TTAAGGATAGCCGAACTTTTAAGTTACCAGAAGCGACAGAATTTTCAAGT |
| 117 | TTGCCCATAATCAAAATTTTTCACCGGAACCCCCACAAGAATTTTTGAG |
| 118 | GGAAACGCAATGATAGCAGCACCGTAATCAGTAGGAAACCGA |
| 119 | CATCTTTTTTTAGCGTCAGAC |
| 120 | TGTAGCGCTATTAGCGTTTGC |
| 121 | AGCCCCCTGTTTTCATCGGCATTTCGGAACCT |
| 122 | ATTATTCTTGCCCCCTGCCTATTTTCGGTCAT |
| 123 | ACAGTTAAGAAACATGAAAGT |
| 124 | ATTAAGAGAGTGCCCGTATAA |

| | |
|---|---|
| 125 | TGTATCTTTTGGTTTATCAGCGGGTCAGTGCTTTTCTTGAGTAACGCTGA |
| 126 | GACTCCTTTTCAAGAGAAGGAAGCGGAGTGATTTTAATAGAAAGGTTAAT |
| 127 | CGGGGTTTTGCTAAACAACTTTCAACAGTTTCTTAGGATTAG |
| 128 | AGGAGCCTAACAACTAAAGGA |
| 129 | ATTGCGAAAAAAGGCTCCAAA |
| 130 | CTCCAAAATAATAATTTTTTCACGCAAGCGCG |
| 131 | AAACAAAGCCCCCAGCGATTATACTTGAAAAT |
| 132 | ATCTTTGATACAACGGAGATT |
| 133 | TGTATCATCACTAAAACACTC |
| 134 | GTTTACTTTTCAGACGACGATACGAAAGAGGTTTTCAAAAGAATACGCCTG |
| 135 | ATAAATTTTTGTGTCGAAATTCATCAGTTGTTTTGATTTAGGAACCCTC |
| 136 | CTCCATGTTACCATTATTACAGGTAGAAAGATCCGCGACCTG |
| 137 | TATCATAATACCACATTCAAC |
| 138 | TAATGCAGGTAAGAGCAACAC |
| 139 | GAGGCATAATACATAACGCCAAAATTGCTCCT |
| 140 | TTTGATAATAGAGAGTACCTTTAAGGAATTAC |
| 141 | GTCAGGATGAGGTCATTTTTG |
| 142 | CGGATGGCCAAACTCCAACAG |
| 143 | AACGGTTTTTAATCGTAAAACAAAGCGAACCTTTTAGACCGGAAGTTAGA |
| 144 | GCTTAATTTTTGCTGAATATAAGCTGATAAATTTTTAATGCCGGACGATG |
| 145 | TCAACATGTTTAATATGATATTCAACCGTTCTATGCTGTAGC |
| 146 | ATTCGAGCTTCTAGCATGTCAA |
| 147 | GCGTTTTATCATATGTACCCC |
| 148 | GGTTGATAGACTTCAAATATC |
| 149 | TAAGATTTTGAAGCCCGAAAATCAGAAAAGTTTTCCCAAAAACAGAAACG |
| 150 | ATGTGAAAATATTTAAATTGTGAAGATTGTATAAGCGCGAGT |
| 151 | TTGCAGCAGGGAACAAACGGC |
| 152 | GGATTGACGGCCCTGAGAGAG |
| 153 | CGTAATGGGATCTTCACCGCCT |
| 154 | ATTAAAACAACCCGTTTTTGGATTCTCCGTAGCGGTCCACTTTTCTGGTT |
| 155 | TGCCCTCCGAAATCGTTTTGCAAAATCCCTGCCAGCTTTCTTTTATCAAC |
| 156 | TCTATCCTGTTTGATGGTGGTCAGCAGGCGAAAATCAGGGCG |
| 157 | TGATTTTTAGCCCTAAAACACGTCAAAGGTTTTGCGAAAAACCGATGGC |
| 158 | CCACTTTTTCGTGAACCATCTAAGAATACGTTTTGGCACAGACAAGCGAAC |
| 159 | TGAAAGCGACCCAAATCAAGT |
| 160 | TTTTTGGGAACCCTTCTGACC |
| 161 | GTCGAGGTGCCAACAGAGATAG |
| 162 | AATGAAATTAGTCTTTAATGCTATTTTTGAATGGCTAAATCT |
| 163 | GAATCACGCTGAGAATTTTGAGTCAATAGTCCACGCTGAGTTTTAGCCAG |
| 164 | CAGCAAAAGCATCACTTTTTTGCTGAACCTTAATTAATTTTTTTCCCTTA |
| 165 | GTCGCTATCAAATATCAAACC |
| 166 | CTCAATCAGCTTCTGTAAATC |
| 167 | ATATCTGGTCATGAATAACCTT |

| | |
|---|---|
| 168 | AGAACGATAGCTTAGATTAAGCTTGAAAACATAGCGCGAGAA |
| 169 | GACAAAACTTTTTCATTTTATATATTTTAGATCCTGAATCTTTTTACCAA |
| 170 | CGCTAGTTACAAAATTTTTAAACAGCCATAGCAAATCCAATTTTTCGCAA |
| 171 | ACAATTTTTTAATTTCATCTT |
| 172 | CTGACCTATTTGCACCCAGCT |
| 173 | AATTTAATGGTTTAGTTGCTAT |
| 174 | GGGAGAGAGCCTAATTTGCCAACGAGCGTCTTTCCAATTAAC |
| 175 | TAGACTGAACACCCTTTTTAACAAAGTCAGCGCCACCCTCTTTTGAACCG |
| 176 | CCACCAGAACCACCATTTTCCAGAGCCGCCAAACAGGGAATTTTGCGCAT |
| 177 | CTCAGAGCAGGGTAATTGAGC |
| 178 | GCTAATATCGGAACCGCCTCC |
| 179 | CAGAGAGATAAAGAGCCACCAC |
| 180 | CCGTTCCTCAGAGCCGCCACCCTCAGAGCCACCACCCAGTAA |
| 181 | ATTTAGCGTCATACATTTTGGCTTTTGATGATACCGATAGTTTTTGCGCC |
| 182 | GACAATATATTCGGTTTTTCGCTGAGGCTTAAAGCGCAGTTTTTCTCTGA |
| 183 | ACAGCTTGATACAGGAGTGTA |
| 184 | CTGGTAATGTGAATTTCTTAA |
| 185 | AAGTTTTAACGTTGCTTTCGAG |
| 186 | TTTCATCCCACGCATAACCGATGACAACAACCATCGGAGGAA |
| 187 | GCGGATTTTATCGTCATAAAGGCTTTGAGGTTTTACTAAAGACTTGTTTC |
| 188 | CATTATTTTACGGGTAAAATGAAGTTTTGCTTTTAGAGGGGGTAAATACT |
| 189 | TTGCAAAAACGTAATGCCACT |
| 190 | ACGAAGGCTAGCGAGAGGCTT |
| 191 | ACCAACCTAAAAAAAACCAAAA |
| 192 | GATTGCACTGGATAGCGTCCATAGTAAAATGTTTAGATCAAA |
| 193 | TTAATTTTTATTTTGTTAAAATTATAGTCATTTTGAAGCAAAGCGAAGAT |
| 194 | ATAGGGTTATCAAAAATAATTTGTTGAGACTGAGTGAGC |
| 195 | CGCGTCTGAGAATAGCCCGAGTGTTGAGACTGAGTGAGC |
| 196 | GCCTTCCTGTATATAAATCAAA |
| 197 | GGAACGCCGAGTGTTGTTTTTTTCCA |
| 198 | GTTTGGAACAGAAGATAATTTTTAAC |
| 199 | AGAGGTGACCTTTTTAACTTTTTCTC |
| 200 | ACCACCAGCAAGAGTCCACTATGTTGAGACTGAGTGAGC |
| 201 | TTAAAGAAAAATACCGAACGATGTTGAGACTGAGTGAGC |
| 202 | CGTGGACTCCAATCGCCATTAA |
| 203 | AGAGACTAGGCGGTCAGTATTTGTTGAGACTGAGTGAGC |
| 204 | AACACCGCAATCATAGGTCTGTGTTGAGACTGAGTGAGC |
| 205 | CTGCAACAGTGGAATTTATCAA |
| 206 | CGGCTTAGTTTGTTTAACTTTTTGTC |
| 207 | AAAAATGAACGATTGGCCTTTTTTTG |
| 208 | AACGATTTGTTGGGTTATATATGTTGAGACTGAGTGAGC |
| 209 | ACTATATGAATCCAAATAAGATGTTGAGACTGAGTGAGC |
| 210 | TAAATGCTGATTTATTTATCCC |

| | |
|---|---|
| 211 | CAGGTCAGAAATAGCAGCCTTTGTTGAGACTGAGTGAGC |
| 212 | TACAGAGACAGGAGGTTGAGGTGTTGAGACTGAGTGAGC |
| 213 | GAATAACATAAGCCAGCATTGA |
| 214 | CGGGATCGAAACAAATAAATCTGTTGAGACTGAGTGAGC |
| 215 | CTCATTAAAAGGCCGCTTTTGTGTTGAGACTGAGTGAGC |
| 216 | AGCCAGAATGGGCAGGGAGTTA |
| 217 | ATATTCACTCACCCTCAGTTTTTCAG |
| 218 | CGAAAGACAGTTCAGAAATTTTTACG |
| 219 | CTTTAAACAGCATCGGAACGATGTTGAGACTGAGTGAGC |
| 220 | GGGTAGCATCCCCCTCAAATGTGTTGAGACTGAGTGAGC |
| 221 | ACGGCTACAGATATTCATTGAA |
| 222 | AGAATGACTTTTTAACCATTTTTATA |
| 223 | CAGCTCATCATAAATCAAAAATGTTGAGACTGAGTGAGC |
| 224 | TCAGGTCTATTTTTGTTAAATTGTTGAGACTGAGTGAGC |
| 225 | TTACCCTGACTATTCGCATTAA |
